# Altered Antibiotic Transport in OmpC Mutants Isolated from a Series of Clinical Strains of Multi-Drug Resistant *E. coli*

**DOI:** 10.1371/journal.pone.0025825

**Published:** 2011-10-28

**Authors:** Hubing Lou, Min Chen, Susan S. Black, Simon R. Bushell, Matteo Ceccarelli, Tivadar Mach, Konstantinos Beis, Alison S. Low, Victoria A. Bamford, Ian R. Booth, Hagan Bayley, James H. Naismith

**Affiliations:** 1 Centre for Biomolecular Sciences, University of St Andrews, St Andrews, United Kingdom; 2 Chemistry Research Laboratory, Department of Chemistry, University of Oxford, Oxford, United Kingdom; 3 School of Medical Sciences, University of Aberdeen, Institute of Medical Sciences, Aberdeen, United Kingdom; 4 Department of Physics and IOM/CNR UOS SLACS, University of Cagliari, Monserrato, Italy; University of Cambridge, United Kingdom

## Abstract

Antibiotic-resistant bacteria, particularly Gram negative species, present significant health care challenges. The permeation of antibiotics through the outer membrane is largely effected by the porin superfamily, changes in which contribute to antibiotic resistance. A series of antibiotic resistant *E. coli* isolates were obtained from a patient during serial treatment with various antibiotics. The sequence of OmpC changed at three positions during treatment giving rise to a total of four OmpC variants (denoted OmpC20, OmpC26, OmpC28 and OmpC33, in which OmpC20 was derived from the first clinical isolate). We demonstrate that expression of the OmpC K12 porin in the clinical isolates lowers the MIC, consistent with modified porin function contributing to drug resistance. By a range of assays we have established that the three mutations that occur between OmpC20 and OmpC33 modify transport of both small molecules and antibiotics across the outer membrane. This results in the modulation of resistance to antibiotics, particularly cefotaxime. Small ion unitary conductance measurements of the isolated porins do not show significant differences between isolates. Thus, resistance does not appear to arise from major changes in pore size. Crystal structures of all four OmpC clinical mutants and molecular dynamics simulations also show that the pore size is essentially unchanged. Molecular dynamics simulations suggest that perturbation of the transverse electrostatic field at the constriction zone reduces cefotaxime passage through the pore, consistent with laboratory and clinical data. This subtle modification of the transverse electric field is a very different source of resistance than occlusion of the pore or wholesale destruction of the transverse field and points to a new mechanism by which porins may modulate antibiotic passage through the outer membrane.

## Introduction

Multi-drug resistance is increasingly prevalent in clinically important pathogens and threatens to hinder treatment of diseases hitherto thought “cured”. Bacterial resistance occurs by a combination of a reduction in drug passage through the outer membrane into the cell, modification of drug targets (protein or the metabolic pathway), antibiotic degradation or increased transport of antibiotic out of the cell. The outer membrane of Gram negative bacteria presents a formidable barrier to polar molecules. Porins, which are beta-barrel proteins that traverse the bacterial outer membrane, facilitate the uptake of small polar nutrients and are the main entry pathway for many antibiotics [1], [2]. Several clinical studies have linked antibiotic resistance to altered expression of porins, or their restricted function due to point mutation(s) (reviewed in [3]). The selection pressure for such mutants must balance increased resistance against the loss of capacity for nutrient access to the periplasm.

*E. coli* produces two major porins, OmpF and OmpC [2]. OmpC has a smaller effective channel than OmpF [4], [5] and is preferentially expressed in higher osmolarity environments, such as the human gut [2]. Many antibiotic-resistant strains of *E. coli* have reduced or no expression of OmpF [6], [7] and this has been taken as evidence that OmpF is the primary pathway for antibiotic import [2]. More recently, it has been argued that the role of OmpC in antibiotic resistance has been underestimated [3], [8]. OmpC mutations have been detected in antibiotic resistant *E. coli* strains isolated after antibiotic treatment [9], [10] as well as in similarly derived *Enterobacter aerogenes* where mutations in an OmpC homolog (Omp36) have been reported [9].

**Figure 1:**
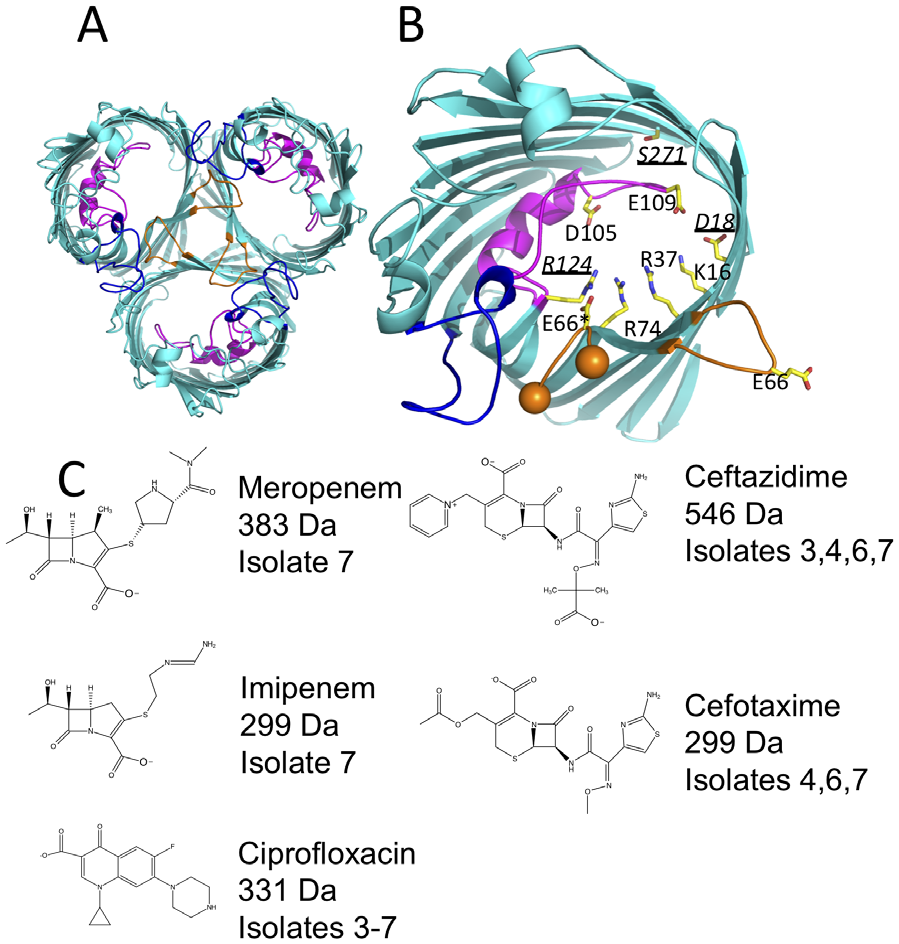
Structure of OmpC. (a) The trimer of OmpC20 viewed from the outside the cell, loop L2 (orange) reaches across from one monomer into the pore of another making three salt contacts per trimer, L3 (magenta) forms the constriction zone and L4 (blue) contributes to trimer stability and forms the entrance to the pore. (b) The constriction zone of OmpC20, the color scheme is as Fig. 1a. The side chains (carbon yellow, oxygen red, nitrogen blue) of the charged residues at the constriction zone are labeled. The residues that are mutated in going from OmpC20 to OmpC33 are underlined and shown in italic. Two of the three residues that are mutated in OmpC33 sit at the constriction zone, the third *S271* sits behind L3. Residue E66* from loop L2 from another subunit (shown with orange spheres at termini) reaches into the pore the salt bridge with *R124*. (c) The structure, charge and molecular weight of the beta-lactam antibiotics. The number of the isolates in which clinically significant resistance occurs are noted [10]. Isolates 1, 2 and 3 contained OmpC20, isolate 4 OmpC26, isolate 5 OmpC28 and isolates 6 and 7 OmpC33.

The crystal structures of OmpF [11] and OmpC [12] show that they are both built of 16 amphipathic β-strands arranged into a barrel with a central pore. The strands are connected by long extracellular loops (denoted L1, L2 etc) and short periplasmic turns (Fig. 1a). Both proteins are found as trimers that are held together by hydrophobic interactions between the barrel surfaces and by the loop L2, each of the three L2 loops in the trimer reaches into the adjacent monomer forming a conserved salt bridge between L2's glutamic acid and an arginine inside the barrel [11]–[13] (Fig. 1a). L3 folds into the channel and narrows the pore at about the half way point, forming a “constriction zone” or “eyelet” that governs the effective pore size [11], [12]. At the eyelet, there is a strong transverse electric field due to the presence of conserved basic amino acid residues on the barrel wall facing acidic residues on L3 (Fig. 1b). Due to conservation of the critical residues this transverse field is found in all OmpC and OmpF porins and it is proposed to govern ion selectivity and channel permeability to polar molecules [14]. Zwitterionic antibiotics are thought to bind at the entrance to the pore and interact with these charged residues, which facilitates their translocation [15]–[17]. Blockage of the OmpF porin by various zwitterionic antibiotics (e.g. penicillin) has been observed at high concentrations and attributed to the binding events that precede their translocation [18], [19]. Constructed mutations of residues located at the constriction zone of OmpF and other porins that alter the passage of small molecules, including antibiotics, have been extensively studied (reviewed in [3], [8]). In many of these cases, the resistance was caused by radical alteration to the hydrogen bond network that constitutes the transverse charge field surrounding the pore eyelet, not simply reduction of the pore size.

Detailed studies of clinically-resistant bacteria with modified OmpC are relatively rare, but as the role of OmpC in antibiotic transport becomes clearer the importance of such research grows [8]. One study described a series of OmpC mutants isolated from a patient with Caroli syndrome who had chronic *E. coli* infection of liver cysts [10]. Seven *E. coli* isolates were collected, either from blood samples or directly from liver abscesses over a two-year time period [10]. Measurement of the minimum inhibitory concentrations (MIC) for each of the prescribed antibiotics revealed that the *E. coli* isolates had progressively greater antibiotic resistance (Fig. 1c). However the MIC values fluctuated from isolate to isolate for particular antibiotics and there was no simple monotonic increase in MIC against all antibiotics [10]. The authors noted the *E. coli* isolates lacked OmpF expression and that there were three sequential cumulative changes in the OmpC protein [10]. The overall sequence of the OmpC protein from the first isolate (OmpC20) is quite different from that found in the lab strain *E. coli* K12 and it is thought that the infecting organisms evolved from the uropathogenic *E. coli* strain O6, which expresses a related OmpC protein (Fig. S1). The subsequent isolates retain this sequence but have also acquired further changes: at D18E (OmpC26), D18E S271F (OmpC28) and D18E, S271F, R124H (OmpC33). Interpretation of the clinical data is complicated, as other changes in the strains occurred in parallel, including alteration of expression levels of other proteins, notably ß-lactamases and in the last two strains OmpC expression itself was decreased. We have combined structural studies with biological and *in silico* simulations to investigate the mechanism by which the porin mutations contribute to antibiotic resistance. These data support a model in which perturbation of the transverse electrostatic field by mutation, rather than steric occlusion of the pore, is a major contributor to modified antibiotic transport through the porin.

## Results

### A role for OmpC mutations in perturbing transport into *E. coli* cells

**Figure 2:**
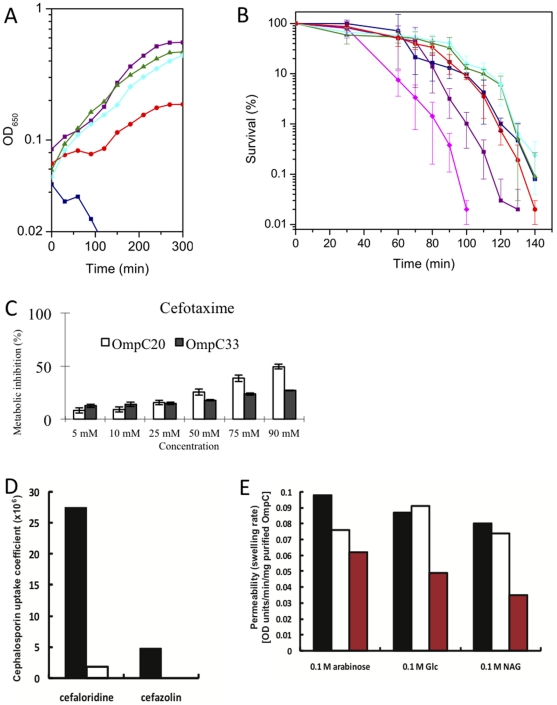
Evidence that mutation in OmpC affects cell viability. (a) LB growth curve: Δomp9 cells transformed with the empty vector (navy squares), OmpC20 (purple squares), OmpC26 (green triangles), OmpC28 (cyan diamonds) and OmpC33 (red circles) were grown in LB medium to mid-exponential phase and then diluted 1 in 10 into 15% diluted LB medium to give a final 25% strength LB culture. The Δomp9 cells transformed with OmpCK12 are not shown as they followed the OmpC20 curve (see Fig. S3a). A representative example of three replicates is shown. (b) Antibiotic inhibition of viability: Viability assays were performed with Δomp8 cells transformed with the empty vector pHSG575, OmpCK12, OmpC20, OmpC26, OmpC28 and OmpC33 (colours and symbols as (a) except OmpCK12 (magenta diamonds))) treated with 10 µg ml^−1^ cefotaxime at t = 0. The mean ± SD is shown (see also Fig. S3). (c) Metabolic inhibition by cefotaxime of HN705 cells expressing OmpC20 and OmpC33. A bar chart shows the extent of metabolic inhibition in the presence of antibiotics. The precise values obtained in this experiment varied but the trend was consistent (OmpC33 more resistance than OmpC20). Figure S8 shows a divergent set of data. (d) Antibiotic penetration was also determined by the relative rate of β-lactam (cephaloridine and cefazolin) hydrolysis in clinical isolate 1 which expresses OmpC20 (black) and clinical isolate 7 which expresses OmpC33 (white). (e) Liposome swelling assay. The rates of permeation of arabinose, glucose and N-acetylglucosamine into phosphatidylcholine liposomes reconstituted with OmpCK12 (black), OmpC20 (white) and OmpC33 (red) were measured using the liposome swelling assay [4].

To establish whether there was a role for the OmpC function in altering the MIC for different antibiotics clinical isolates were transformed with a plasmid expressing OmpC K12. These data show a drastic reduction in the MIC (Table 1) for each strain when OmpC K12 is expressed in the clinical isolates. We infer that OmpC mediated transport of antibiotics into the cell is an important component of clinical resistance. (As a control we tested gentamicin resistance, which does not require transport through the porin to the periplasm and the MIC was unchanged by the presence OmpC.) Our purpose is not to compare OmpC K12 with OmpC20. OmpC K12 comes from a lab strain, as a result the evolutionary processes lead to its current sequence are different. The purpose of the experiment was to determine whether transport of antibiotics into the clinical strains is a factor in the observed resistance. The clinical isolates however exhibited variations in porin (and other protein) expression that could determine resistance. Thus, to deconvolute these confounding factors (differential expression of OmpC and varying cellular background) from any modification of transport properties that accompany the change from OmpC20 to OmpC33, we undertook a number of different biological assays of porin function under conditions where expression levels and cellular background were controlled. We chose three different isogenic strains that lack functional porins (Δ9Omp and Δ8Omp) [20] and HN705 [21] to isolate the effects of the three porin mutations from other factors. Δ9Omp cells were transformed with low copy number plasmids expressing either OmpC K12 (as a control) or one of the mutant OmpC proteins derived from the clinical isolates. All of the porins expressed to the same level within the limits of our detection (Fig. S2). Porins are predicted to become rate limiting for growth when the nutrient concentration is lowered [22]. No difference in growth rates was observed between transformants, incubated in full strength LB and thus, growth of the strains was assessed in diluted LB media (diluted to 25% V/V with sterile H_2_O). A clear differentiation between the parent strain transformed with the empty plasmid (pHG575) and the strains expressing functional porins was observed (Fig. 2a). The Δ9Omp strain failed to grow, whereas all the strains expressing an OmpC protein grew confirming all OmpC mutants were functional. The fastest growth was observed with the OmpC K12 (similar rates observed for OmpC20, OmpC26 and OmpC28) and the slowest with OmpC33. The data clearly show that OmpC33 has different transport properties from OmpC20 (OmpC26, OmpC28) which is consistent with OmpC33 offering a diminished route of entry for small organic molecules, such as the amino acids that are the major nutrients for growth in broth [23], [24] (Fig. 2a).

**Table 1:** Reduction of MIC by co-expression of OmpC K12 in the clinical *E. coli* isolates.

Isolate	20		26		28		33	
pOmpC K12	−	+	−	+	−	+	−	+
Cefotaxime	0.25	0.06	>32	4	>32	4	>32	32
Imipenem	0.25	0.12	1	0.5	1	1	8	0.5
Gentamicin	1	1	1	1	1	1	2	2

Antibiotic susceptibility testing was performed using M.I.C. Evaluator strips in Mueller-Hinton broth. The following antibiotics were studied: cefotaxime, imipenem and gentamicin. MIC values given are µg ml^−1^. Clinical isolates were tested in parallel with these same isolates transformed with a plasmid a plasmid carrying the *ompC K12* gene.

A similar differentiation between transport properties for the porin variants was obtained from an analysis of antibiotic-mediated cell killing. In the presence of cefotaxime (2 µg ml^−1^ in MHB medium) *E. coli* Δ8Omp cells transformed with OmpC K12 were killed significantly faster than those expressing the clinical mutants (Fig. 2b). In this assay, it is OmpC20 that is significantly more sensitive to cefotaxime than the other clinical OmpCs. However this pattern appears to be antibiotic-specific, since cells expressing this OmpC33 actually exhibited increased sensitivity to ceftazidime (relative to OmpC20, 26, 28 and even K12); no change in imipenem sensitivity was observed (Fig. S3). Cells with empty plasmid were the least sensitive, consistent with OmpC being the route of antibiotic entry into these cells. As expected gentamicin sensitivity was unaffected by the identity or presence of OmpC, as this antibiotic has a different mode of action and entry (Fig. S3d). Thus the changes in sequence between OmpC20 and OmpC33 do differentially modify the sensitivity of lab strain *E. coli* Δ8Omp to antibiotics. As an additional experiment, sensitivity to cefotaxime was tested in HN705 cells after complementation of the porin deficiency with either OmpC20 or OmpC33 using the integrated cell metabolic activity assay based on MTT (3-[4, 5-dimethylthiazol-2-yl]-2, 5-diphenyl tetrazolium bromide) reduction by viable cells [25]. Expression levels of the porin were identical and thus did not affect the assay outcome (Fig. S2). When metabolic activity is reduced by addition of an antibiotic, less formazan is produced [25]. HN705 cells expressing OmpC20 produced consistently less formazan in the presence of cefotaxime than HN705 cells expressing OmpC33 (Fig. 2C). This indicates a reduction in the effect of cefotaxime upon the metabolic activity of cells expressing OmpC33 consistent with the decreased transport relative to cells expressing OmpC20.

Antibiotic penetration was also determined by the relative rate of β-lactam (cephaloridine and cefazolin) hydrolysis using the classical Zimmerman and Rosselet protocol [26]. The permeability of the porin in whole cells is determined by dividing the rate of hydrolysis of β-lactam in intact cells by the rate of hydrolysis of lysed cells [5]. We conducted this assay with the original clinical isolates that display differing complements and levels of β-lactamase activity. By using this ratio, the process normalizes for this variation in activity of the endogenous complement of β-lactamase enzymes thus allowing comparison between strains where the intrinsic β-lactamase activity may vary. The rate of penetration of the cephaloridine decreased 20-fold for the clinical isolates expressing OmpC33 relative to OmpC20 and cefazolin uptake was undetectable in isolates expressing OmpC33 (Fig. 2d).

Finally, the permeability of the porins OmpC20 and OmpC33 to sugars was assessed using the purified proteins reconstituted into phosphatidylcholine proteoliposomes, using OmpC K12 as a reference [4]. Carbohydrate transport through the porin into the liposomes causing them to swell due to water movement into the liposome lumen. Thus over a fixed time period, the increase in size is related to the rate of transport of sugar into the liposome. Significantly slower entry of sugars (arabinose, glucose and N-acetylglucosamine) was observed into liposomes containing OmpC33 compared to those containing OmpC20 (the entry rate for OmpC20 differs little from OmpC K12) (Fig. 2e).

### Ion flow through different OmpC mutants

**Figure 3:**
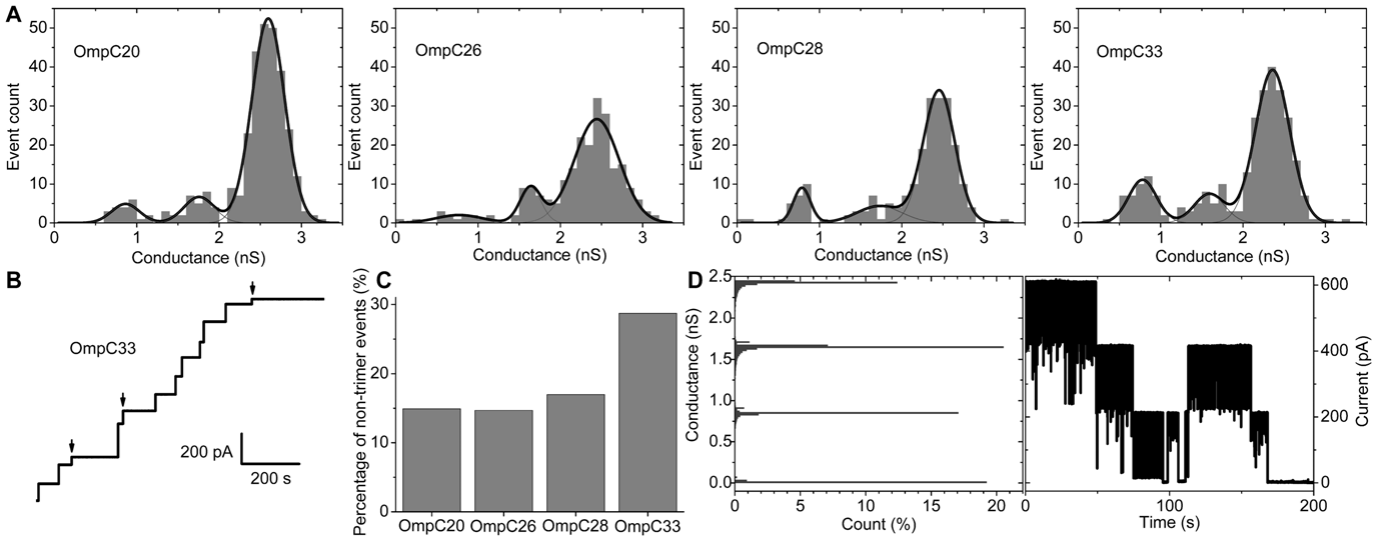
Single channel measurement on OmpC proteins. (a) Distribution of conductance values from individual insertions obtained from current recordings, in 1 M KCl, 10 mM Tris, pH 8.0, at +50 mV. Three Gaussian peaks with freely varying centres and widths were fitted to each conductance histogram. (b) Typical consecutive channel insertions of OmpC33 measured in 1 M KCl, 10 mM Tris, pH 8.0, +50 mV, 100 Hz low-pass filter. Arrows indicate non-trimeric insertions in this segment. (c) Percentage of individual insertion steps that were not trimers for each OmpC mutant studied. (d) Typical trace of voltage gating in a single OmpC20 trimer, and corresponding all-point conductance histogram recorded in 1 M KCl, 10 mM Tris, pH 8.0, +250 mV, 1 kHz low-pass filter.

We performed a series of single molecule conductance measurements on each OmpC mutant to determine whether the changes observed above were replicated in the movement of small ions, which would indicate a major change in pore diameter. We observed a broad range of conductance values ranging from ∼0.6 nS to ∼3.0 nS for all the OmpC mutants (n≥200), which can be fitted to three Gaussian distributions. The peak conductance values were in the ratio 1∶2∶3. Voltage-dependent closure (*vide infra*) indicates the three peaks correspond to insertions with one, two or three open channels. Thus the four mutants had a mean trimer conductance between 2.4±0.4 nS and 2.6±0.4 nS (Fig. 3a). The conductance is similar to the approximately 2.5 nS reported for OmpC K12 in the same electrolyte [27], [28].

Continuous channel insertions producing quantized jumps in conductance measured at +50 mV (Fig. 3B) show that for OmpC33 significantly more insertions fall into the lower two (“monomeric” and “dimeric”) conductance steps than for any of the other three mutants (Fig. 3C).

OmpC protein has previously been shown to undergo the voltage-dependent gating [29]. At voltages above ±220 mV, all mutants undergo a three-step closure reducing the conductance to near zero, a process we conclude reflects sequential closure of each monomer in the trimer (Fig. 3d). These data indicate that there are no major changes in either ion conduction or general porin properties and thus that the changes in antibiotic and sugar transport are a consequence of more subtle alterations in structure. Thus we determined the structures of the different mutant porins.

### Structures of the OmpC mutants

Although the structure of OmpC K12 was known [12] the large number of sequence changes (including insertions and deletions) between it and the clinical mutants, made, in our judgment, *in silico* modeling of OmpC20 unreliable and as a result any functional inference drawn from such a model would lack robustness. Therefore, we experimentally determined the crystal structures of five OmpCs (OmpC20, OmpC26, OmpC28, and OmpCO6) at resolutions ranging from 2.3–2.85 Å but we were only able to obtain a 3.55 Å resolution data set for OmpC33 (Table 2). We were unable to obtain higher quality data for OmpC33; this variant seems more prone to aggregation and gave poor quality crystals. All structures are trimeric and share the characteristic porin 16-stranded antiparallel β-barrel [11], [12]. The structures superimpose with an average root mean square deviation (r.m.s.d.) of 0.3 Å for all Cα atoms (Fig. 1a). The β-strands are connected by 8 short turns (2–8 residues in length) on the periplasmic side and 8 long loops (L1 to L8 of 9–30 residues in length) on the extracellular face. L3 folds back into the lumen of the barrel forming the constriction zone and the trimer interface is formed by the packing of hydrophobic residues on the outer edges of strands β1 to β5 and further stabilized by L2 which reaches into the neighboring monomer. In the trimer, the outside surface resembles a bowl with the walls of the bowl formed by the loops 1, 4, 5, 6, 7 and 8 from each monomer.

**Table 2:** X-ray data statistics of OmpC structures.

Data collection					
Dataset	OmpCO6	OmpC20	OmpC26	OmpC28	OmpC33
ESRF beamline	ID14.1	ID14.3	BM14	ID14.1	ID14.3
Wavelength (Å)	0.934	0.931	0.976	0.934	0.931
Space group	P6_3_22	C2	P1	P6_3_	P2_1_2_1_2_1_
Unit cell dimensions					
a, b, c (Å)	120.4, 120.4, 158.1	128.0, 74.2, 133.5	74.2, 93.7, 115.9	103.5,103.5, 186.5	142.8,159.7, 164.6
α, β, γ (°)	90, 90, 120	90, 124.75, 90	98.7, 108.8, 109.7	90, 90, 120	90, 90, 90
Resolution (Å)Highest shell	47-2.5(2.6-2.5)	110 - 2.5(2.6-2.5)	29- 2.28(2.34-2.28)	49–2.85(2.9-2.85)	53 - 3.5(3.7-3.5)
Rmerge (%)	10.5 (51.6)	11.7(63.2)	8.4(45.2)	11.8(74.2)	10.8(42.7)
Completeness (%)	99.5(99.5)	99.4(96.7)	97(96);80(20)*	96(97)	85(73)
I/σI	14.3(3.4)	13.1(1.7)	9.9(1.8)	15.5(2.8)	7.1(1.8)
Redundancy	9.2(9.2)	3.6(3.0)	2.2(2.1)	8.1(8.1)	4.0(3.7)
Refinement					
Resolution range (Å)	50-2.5	50-2.5	30-2.28	50-2.85	30-3.5
No. of unique reflections	24046	35609	118130;95882*	25450	40939
Rwork/Rfree (%)	19.1/21.8	21.7/25.7	22.6/26.7	21.5/23.4	26.1/29.3
R.M.S deviations					
Bond length (Å)	0.005	0.006	0.011	0.006	0.007
Bond angles (°)	0.88	0.99	1.36	0.96	0.95
Ramachandran outliers	0	0	0	0	18 (0.9%)
Molprobity (score/centile) [61]	1.3/100	1.7/100	1.9 (94)	1.7 (100)	2.4 (99)
PDB code	2xe1	2xe2	2xe5	2xe3	2xg6

*These data are anisotropic, the completeness statistics relate to the data after manipulation to remove weak or absent measurements (see methods), the untruncated data and truncated data have been deposited.

The overall structure is very close to that reported for the OmpC K12 structure [12], with an alignment of 0.44 Å r.m.s.d. for 340 Cα atoms (Fig. S4). The majority of single residue differences between the two homologs are located in the extracellular loop regions: L2, L4 and L5. The sequence insertions in L4 and L5 have resulted in modified structural arrangements of these loops in OmpC K12 (Fig. S4a,b). There is a cluster of substitutions in L2 (which forms the major trimer interface) but these changes do not affect its overall conformation. The sequence of the L3 constriction loop is highly conserved in all OmpC homologs, with the only difference being G116 of OmpC K12 being substituted for an aspartic acid (D116) in OmpC20. The conformation of L3 is virtually identical in both proteins. Remaining substitutions are limited to several residues in beta-sheets with membrane-exposed side-chains. The residues in OmpC20 that this study highlights as being responsible for the altered transport of antibiotics when mutated (D18, R124 and D273) are all retained in OmpC K12. Examination of the electrostatic surface calculated using CCP4MG shows that OmpC20 has a more negatively charged entrance than either OmpC K12 or the presumed parent OmpC O6 (Fig. S5). Interestingly, the appearance of the electrostatic surface is closer to OmpC K12 than the presumed parent OmpCO6, despite the greater sequence differences. This is reflected in the slightly higher r.m.s.d of 0.49 Å for 343 atoms when superimposing OmpCO6 and OmpC20. In the clinical OmpC mutants, L4 is shorter however interactions of L4 with L1 are conserved. In OmpC20 a transverse electrostatic field is formed by K16, R37, R74, R124 on one side with D18, D105, E109 on the opposite face, the identical transverse field is seen in OmpC K12 [12] and a very similar one in OmpF [11]. The narrowest point at the constriction zone, as defined by HOLE [30], has a radius of 2.9 Å in all structures (Fig. S4c). Examining the pathway of the imaginary sphere used by HOLE [30] to measure radius, reveals that the largest differences between OmpC20 (and 26, 28, 33) and OmpC K12 (and OmpCO6) occur in extracellular cavity above the constriction zone where the channel radius is narrowed by over 1 Å in OmpC20 (and 26, 38, 33), due to changes in L3 and L4 (Fig. S4c).

**Figure 4:**
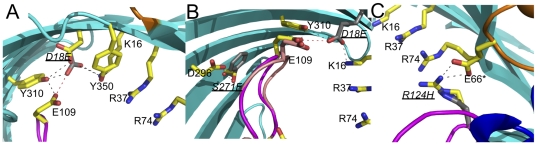
Differences in OmpC structure caused by sequential mutation. (a) Close up of the first mutation site D18E in OmpC26. Residues are colored as Fig. 1b, except for the mutated residue (18E), which is shown with dark grey carbon atoms. The mutation disrupts the hydrogen bond network of D18 (which is shown with yellow carbons) and shifts one of the negative charges that forms the constriction zone at the active site. The larger glutamic acid creates an unfavorable interaction (red dotted line) with E109. There are no other significant changes in structure. The orientation is looking towards the periplasm from extracellular space, the positively charged cluster of residues on one side of the constriction zone are shown and labeled. (b) Close up of the second mutation S271F, which along with D18E occurs in OmpC28. The larger hydrophobic phenyl group (labeled in italic and shown with carbons colored grey) disrupts the hydrogen bonding network of S271 (shown for comparison) and causes a change in the position of the constriction zone loop L3 (shown in salmon with E109 shown with carbons colored carbon and labeled) relative to OmpC26 (the loop is colored purple with E109 in sticks and carbons colored salmon). Other atoms are coloured as Fig. 4a. The orientation is looking towards the periplasm from extracellular space, the positively charged cluster of residues on one side of the constriction zone are shown and labeled. (c) Close up of the site R124H, which occurs in OmpC33 along with D18E and S271F. The colour scheme is as Fig. 4a. The position of 124H (labeled in italic and shown with grey carbons) is not accurate due to the low resolution of the OmpC33 structure but the salt contact of R124 (shown for comparison with carbons colored yellow) with E66* (from the neighbouring subunit) which is important for trimer stability will be disrupted. The mutation will also perturb the electrostatic field by removing a positive charge. The orientation is looking towards the periplasm from extracellular space, the other positively charged cluster of residues on one side of the constriction zone are shown and labeled.

The mutations observed in going from OmpC20 to OmpC33 cluster around the constriction zone. In OmpC26 (D18E), the longer side chain of glutamic acid protrudes into the lumen making a new salt bond to K16 (Fig. 4a). A water molecule bound to the carboxyl group of 18E makes a new hydrogen bond network with E109 and Y310. The presence of this mutation slightly narrows the radius of the pore on the periplasmic side (below but not at the constriction zone) (3.28 Å to 2.95 Å). This creates in OmpC26 an elongated constriction zone along the axis normal to the membrane. In OmpC26 and OmpC20, a water molecule and S271 are at the centre of a hydrogen bond network (Fig. 4B). In OmpC28, the S217F mutation changes this network and moves the tip of L3 (centred on E109) slightly further (<1 Å) into the pore (but not at the constriction zone). In OmpC33, R124 is mutated to a histidine residue but due to the limited resolution of this structure, we cannot experimentally assign the side chain conformation. We are confident that the main chain is, however, unaffected. In all the other OmpC proteins, R124 makes a salt bridge with E66 (from the neighboring subunit L2) (Fig. 4C); the shorter His residue must disturb the all three inter-subunit salt bridges in the trimer. Thermal dissociation experiments shows that the OmpC33 trimer decomposes into monomers at significantly lower temperatures than the other isolates in two different detergent systems (Fig. S6).

In the highest resolution structure (OmpC26) we can see additional electron density in some of the six monomers, which we modeled as SO_4_^2−^ bound by the basic residue cluster K16, R37, R74 and R124 of the transverse electric field. Docking [15] and molecular dynamics [31] have shown ampicillin bound to this cluster in OmpF.

### Molecular Dynamics

Molecular dynamics (MD) simulations were employed to examine the effect of mutation on OmpC20 structure at ambient temperature. The mutation D18E perturbs the hydrogen bonding arrangement at the pore with its precise effect varying between subunits. In two subunits the mutation slightly weakens the hydrogen bond (salt contact) with K16 (60% vs 21% of simulation time where contact is preserved) and creates a new contact with R37 instead (Table 3). In the other subunit the D18E K16 contact is stabilized (69% vs 41%), as seen in the OmpC26 and OmpC28 crystal structures. The S271F mutation does not appear to influence hydrogen bonds. The R124H mutation disrupts the critical hydrogen bond to the E66 from the loop 2 of the neighboring subunit. Molecular dynamics shows this contact is essentially abolished in two of the three monomers (Table 3). The mutations R124H and D18E perturb the constriction zone, which is manifested in the increased conformational variability of the positive charges (R37, R74 and R124H) in OmpC33 when compared to OmpC20.

**Table 3:** The time that a hydrogen bond is present in molecular dynamics 45 ns simulation of OmpC20 and OmpC33.

		OmpC20	OmpC33
near S271F			
N296(s)	D312(s)	94%	98%
E109(m)	D312(s)	60%	62%
N296(s)*	D312(s)*	95%	99%
E109(m)*	D312(s)*	48%	68%
N296(s)**	D312(s)**	94%	97%
E109(m)**	D312(s)**	57%	49%
near D18E			
K16(s)	D18(s)	65%	-
K16(s)*	D18(s)*	41%	-
K16(s)**	D18(s)**	55%	-
K16(s)	D18E(s)	-	28%
K16(s)*	D18E(s)*	-	69%
K16(s)**	D18E(s)**	-	14%
R37(s)*	D18(s)*	22%	-
R37(s)	D18E(s)	-	81%
R37(s)*	D18E(s)*	-	0%
R37(s)**	D18E(s)**	-	84%
near R124H			
R124(s)	E66(s)**	111%	-
R124(s)*	E66	249%	-
R124(s)**	E66(s)*	91%	-
R124H(s)	E66(s)**	-	0%
R124H(s)*	E66	-	0%
R124H(s)**	E66(s)*	-	122%

100% indicates 1 hydrogen bond is present throughout the simulation, >100% indicates more than one hydrogen bond.

(s) denotes a side chain interaction, (m) a main chain interaction,

*B subunit,

**C subunit.

**Figure 5:**
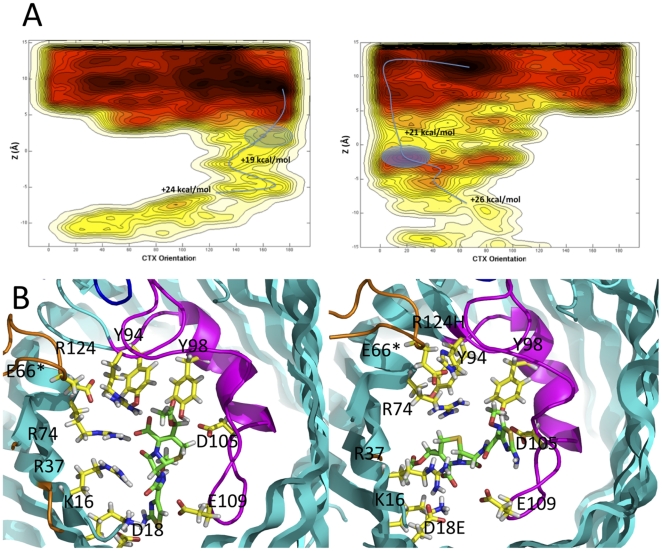
Molecular dynamics analysis of cefotaxime penetration. (a) Free energy surface reconstructed from metadynamics simulations for the translocation of cefotaxime in OmpC20 (left) and OmpC33 (right). Plotted are θ (the orientation of the molecule) on X-axis against Z (distance along the pore). A blue line indicates the lowest energy route from the extracellular space (+ve values of Z, top of the Figure) to the periplasm (−ve values of Z, bottom of the figure). The circled region represents the most likely conformation at the constriction region (Z approximately -2). The color scheme goes from black to white passing through red and yellow to indicate respectively regions with high probability of occupation (minima of the free energy or affinity sites) and with low probability (maxima of the free energy or transition states). Each line represents a difference of 1 kcal/mol in energy. (b) Snapshots extracted from the molecular dynamics trajectory showing cefotaxime at the constriction zone (the circle in Figure 5(a)) of OmpC20 (left) and OmpC33 (right). There is a very prominent difference in the orientation and binding of the cefotaxime at the constriction zones of the two proteins.

Molecular dynamics suggests that the pore area of OmpC33 actually has very a small increase (within fluctuations) in the constriction region compared to OmpC20 (Fig. S4d), consistent with the crystal structures, which showed no difference in pore radius (Fig. S4c). We analyzed the passage of cefotaxime through OmpC20 and OmpC33 using metadynamics (Fig. 5a). The calculations show a lower barrier for transit through OmpC20 than in OmpC33. During translocation through OmpC20, cefotaxime penetrates with the aminothiazoly group down (the θ coordinate in Fig. 5a assumes values near 180°), interacting with an arginine cluster at the constriction zone, (Fig. 5c). This type of interaction with basic residues, has been seen in other porin-mediated antibiotic transport pathways [31] as well in the phosphate-specific transfer through the OprP channel [32]. While on the extracellular side the reconstructed free energy surfaces show the occurrence of all the allowed orientations for cefotaxime in both channels, remarkably it penetrates through the OmpC33 constriction region in the opposite orientation (θ near 0°, as shown in Fig. 5a) and makes no specific interactions with the arginine cluster (Fig. 5b). Instead it occupies the space between R37 and R74, created by the disruptive substitutions R124H and D18E (Fig. 5b). The calculation of the hydrogen bonds and hydrophobic contacts between cefotaxime and the porin at the constriction region shows a few persistent contacts in OmpC20 with contacts tending to be more transient in OmpC33 (Fig. S7).

## Discussion

Retention of porin function is required for virulence – it is severely attenuated in a knockout mutant of the regulator ompR in *S. typhimurium* (eliminating expression of both OmpC and OmpF) [33], and the triple gene knockout *ompC*, *ompF* and *aroC E. coli* strain is an avirulent candidate vaccine [34]. Thus, retention of porin function was likely to be critical for the establishment of the *E. coli* infection of the patient's liver that resulted in the isolates investigated in this study. Changes in outer membrane proteins are recognized to be very important in the development of clinical antibiotic resistance [3], [8] but most reports from the clinic describe diminished porin expression. Several *in vitro* studies have constructed mutations in OmpF (and analogues) that partially block the pore and so decrease antibiotic and small ion permeability (reviewed in [3], [8]). Only one of these, a glycine to aspartate change on L3, has so far been observed in the clinic. It reduces both ion flow and antibiotic permeation [9] consistent with pore occlusion.

Point mutations of porins resulting in clinical resistance have been only rarely characterized but are predicted to be important in defending against carbapenem antibiotics [3]. The series of single site OmpC mutations from *E. coli* that had co-evolved with drug resistance presents a previously unstudied route to clinical resistance [10]. The absence of OmpF in these isolates means that OmpC is not only critical for nutrient acquisition but is also the main pathway for antibiotic entry to the periplasm. The loss of OmpF expression is common in many enterobacteria inhabiting a human host and is presumed to occur because OmpC has a smaller pore, is more suited to higher ionic strength and is in general less permeable to β-lactam antibiotics [3]. Thus if OmpC mutation can be demonstrated to contribute to clinical antibiotic resistance it may represent a more widespread phenomenon, fulfilling earlier predictions [3].

Relying solely on clinical data to evaluate the significance of the OmpC mutants is hazardous since there are many simultaneous and possibly confounding mechanisms [10]. The role of the three porin mutations observed in the clinic in increasing resistance to some antibiotics is supported by a range of analyses in well-defined *E. coli* strains and with the purified proteins. The transformation of the clinical isolates with plasmids expressing OmpC K12 dramatically lowered the MIC for cefotaxime (Table 1), consistent with porin function having a significant role in the overall portfolio of drug resistances. Transforming isogenic porin-deficient *E. coli* with different *ompc* genes from the clinical isolates allowed us to focus on the functional consequences of the OmpC mutations that occur in the clinic. The mutated OmpC proteins are functional, but there are detectable differences in growth rates amongst the mutants under low nutrient conditions that are predicted to increase the importance of porins for nutrient acquisition [22]. Direct measurement of cell survival shows that a porin-deficient strain of *E. coli* transformed with OmpC26, OmpC28 or OmpC33 is, in each case, less sensitive to cefotaxime than the same strain transformed with OmpC20. Measurement of cell metabolism by MTT reduction also indicates that a porin deficient *E. coli* strain transformed with OmpC33 is less permeable to cefotaxime than one transformed with OmpC20. In a cell free (liposome) system, OmpC33 has reduced transport properties compared to OmpC20 for sugars (uncharged polar molecules that are smaller than the antibiotics) (Fig. 2f). Transit of imipenem was however insensitive to the sequence of OmpC, confirming as we expected that in the clinic there are other factors involved in resistance. Interestingly the change in sensitivity to cefotaxime (OmpC20 to OmpC26) correlates with the introduction of this antibiotic into the regime prior to the isolation of strain 4 (the isolate in which OmpC26 was first detected) [10]. The change in antibiotic sensitivity is not a blanket reduction against all antibiotics, since ceftazidime is more effective against cells transformed with OmpC33 than any other OmpC variant (Fig. S3).

In summary, a divergent set of experiments paints a compelling picture that the three mutations observed in changing from OmpC20 to OmpC33 significantly alters the passage of molecules through the porin. Further, these changes have measurable outcomes on the resistance of isogenic clinical *E. coli* strains to antibiotics. We do not address the differences between OmpC20 and OmpC K12. OmpC K12 was used only as a positive control in our assays and was not found in the clinical study. Our focus was to identify any correlation between antibiotic resistance and the three sequence changes in OmpC that occurred in the clinic. Interpreting the multiple changes in sequence and the different evolutionary history of OmpC K12 and OmpC20 (26, 28 & 33), which would be involved in such a comparison, are beyond the scope of this present study.

The simplest method of engineering resistance is to disrupt the pore size, seen in G112D mutation in Omp36, an OmpC analogue in *Enterobacter aerogenes* [9], [35] which shows a significantly decreased ion conductance. By contrast, single channel measurements do not show any significant change in the average conductance of OmpC20, 26, 28 and 33 (Fig. 3). It is generally accepted that small ion conductance is correlated with pore size. Experimental structural data and molecular dynamics both suggest that the constriction zone size has not altered significantly between OmpC20 and OmpC33 (Fig. S4c, d). Thus our data show changes in antibiotic sensitivity are not correlated with any change in small ion conductance and appear to rule out reduction in pore size as the mechanism of transport reduction.

In the absence of evidence for major changes in the pore size, we examined the structures in more detail to identify a basis for the change in transport. The conserved structure of the constriction zone in porins is recognized as key to controlling molecular translocation [11], [12], [18], [36], [37]. The three mutations cluster close to this region, all three residues are conserved in OmpC K12/OmpCO6 and R124 and S271 are conserved in OmpF. The D18E change (OmpC26, 28 and 33) results in an additional salt bridge with K16 in the crystals and molecular dynamics shows the mutation perturbs the hydrogen bonding at the constriction zone (Fig. 4a, Table 2). The S271F mutation (OmpC28, 33) seems to have only a marginal effect on the constriction zone (Fig. S4c). The bulkier phenyl side chain packs against the L3 loop and the L3 loop is as a result slightly displaced (<1 Å) into the channel. The aromatic ring fills a previously open pocket adjacent to the central pore and but has little other effect. Both in the OmpC33 crystal structure and *in silico*, the R124H mutation disrupts the three salt bridges with E66 of loop 2 found in the trimer. Protein thermal stability tests confirm that the OmpC33 trimer does indeed breakdown into monomers more easily than OmpC20 (Fig. S6). This instability may also be reflected in the more frequent simultaneous insertion of only one or two conducting monomers in OmpC33 compared to OmpC20 (Fig. 3C). When the equivalent arginine residue (R132) was mutated to an aspartic acid in OmpF, trimer destabilization also occurred [38]. We do not believe that trimer destabilization is the principal cause of altered permeation for the following reasons; OmpC26 and OmpC28 are stable but are, like OmpC33, less permeable to cefotaxime, OmpC33 is functional (Fig. 2a) and conducts small ions as a fully open trimer (Fig. 3), OmpC33 can be purified and crystallized as a trimer and finally OmpC33 transformants are actually more sensitive to a different antibiotic (Fig. 2B and S3).

Molecular dynamics analyses of the transit of cefotaxime through OmpC20 and OmpC33 shows different barriers and trajectories (Fig. 5) with a higher barrier to transit observed for OmpC33 than OmpC20. Transit is governed by the interactions with the residues that form the transverse electric field; K16, R37, R74, R124 on one side D18, D105, E109 on the other [14], [39], [40]. Shape and charge of the transiting molecule play significant roles and govern their interactions with the porin thus determining transit efficiency [4], [5]. The D18E mutation alters the position of one of the negative charges on one side of the constriction zone and the R124H mutation removes a positive charge on the other side. This change in the spatial distribution of charges at the constriction zone by definition alters the transverse electric field. Interestingly molecular dynamics suggests that the side chains of the charged residues are more flexible in OmpC33. A molecular dynamics analysis shows that cefotaxime adopts a very different orientation making very different interactions with the protein inside the constriction zone of OmpC33 compared to OmpC20 (Fig. 5a). We suggest this underlies the difference in sensitivity to cefotaxime that we observe. We note that cefotaxime has a negative charge and thus may be the most sensitive to changes in the transverse electric field. The large number of polar groups in carbohydrates which require desolvation and stabilization during transit may make these molecules particularly sensitive to these changes, possibly explaining the decreased rate of entry into liposomes containing OmpC33 (Fig. 2e).

The biochemical study of mutations found in clinical strains of pathogens can help to elucidate mechanisms of resistance. We present clear evidence that subtle changes in OmpC observed in clinical isolates of *E. coli* [10] altered antibiotic permeability and thus cell viability. Our analysis shows polypeptide sequence changes perturb the transverse electric field at the constriction zone, but do not, (in contrast to most engineered mutations) reduce the size of the constriction zone or passage of small ions. Simulations show that the changes in charge distribution in OmpC, as a result of mutation, reduce the transit of cefotaxime through the constriction zones. This type of mutation seems to balance retention of porin function against reduction in drug influx. A recent survey of clinically important mutations in a *Pseudomonas aeruginosa* outer membrane protein OprD [41] identifies many mutations of the full length protein. Many (but not all) of these mutations cluster near the constriction zone of the OprD structure [42]. Mutations at positions 120 and 121 in the porin IB of penicillin and tetracycline-resistant *N. gonorrhoeae* strains are located at the constriction zone and (as with the OmpC mutants) do not seem to perturb small ion flow but rather disfavor antibiotic transit [43]. Thus there is evidence from clinical studies that changes around the constriction zone may leave the pore size and ion flow unchanged, presumably preserving cell viability, while selectively perturbing the transit of large polar molecules such as antibiotics into the cell.

## Methods

### Strains

The original strains isolated from the patient were designated 1 to 7 [10]. The porins studied here were derived from the following isolates: OmpC20 isolate 1; OmpC26 isolate 4; OmpC28 isolate 5; OmpC33 isolate 6.

### Cloning, expression, purification crystallization and stability of OmpC

The genes encoding OmpC protein from clinical *E. coli* isolates and *E. coli* O6 were amplified by PCR using the forward primer ompC10F (5′-GTT AGG TGT CGA CTT CGC CAT TCC GC-3′), and the reverse primer ompC11R (5′-AGA ACC GGT AAC TTC AGT AGC GTG GG-3′) and ligated into the expression vector pHSG575 [44]. The sequences of the clones were verified by DNA sequencing. Plasmid pHSG575 containing the variant *ompC* genes was transformed into mutant *E. coli* strains lacking porins [21].

Transformed cultures were grown at 37°C for 18–24 hours in LB media containing 25 µg ml^−1^ chloramphenicol and 0.5% (w/v) glucose. The cells were then pelleted by centrifugation at 6,000×g (4°C) for 15 min, and then lysed by sonication in 50 mM sodium phosphate, pH 7.5, and 50 mM NaCl. Membranes were resuspended in 2% (w/v) sodium N-lauroyl sarcosine and incubated at 20°C for 2 hours, the supernatant was centrifuged at 100,000×g (4°C) for 1 h. OmpCs were solubilized in 0.5% (w/v) 3-(N, N-Dimethylmyristyl-ammonio) propanesulfonate (SB 3.14, Sigma) overnight at 20°C with stirring.

Proteins were applied to a POROS-HQ anion exchange column pre-equilibrated with 50 mM sodium phosphate, pH 7.5, 50 mM NaCl and 0.025% (w/v) SB3.14 and eluted with 1 M NaCl. Fractions containing OmpC were diluted into low salt concentration and applied to the same column again in order to exchange the detergent solution to a mixture of 0.6% (w/v) 2-hydroxyethyloctylsulfoxide (HESO) and 0.1% (w/v) n-octylpolyoxyethylene (OPOE), or 1% (w/v) n-octyl-β-D-glucopyranoside (βOG). OmpC proteins were further purified by gel filtration (Superdex 200, Pharmacia). Protein purity and integrity was checked by SDS-PAGE and by MALDI-TOF.

Protein purified in 1% (w/v) βOG was added to NuPAGE buffer (Invitrogen), and incubated for 10 min at various temperatures (35°C–100°C) then transferred to ice before loading onto NuPAGE 4–12% Bis-Tris gels (Invitrogen) which were run at 200 V for 35 min and stained by Coomassie. Whole cell samples were harvested and resuspended in 20 µl SDS solubilisation buffer (20% (v/v) glycerol 2% (w/v) SDS, 0.12 M Tris-HCl, pH 6.8, 0.5 g L^−1^ bromophenol blue, 20 ml L^−1^ β mercaptoethanol) and heated at 100°C for 10 min then run described above.

Crystals of OmpCO6, OmpC20 and OmpC26 were grown using the detergent mixture of 0.6% (w/v) HESO and 0.1% (w/v) OPOE at 20°C. Both OmpCO6 and OmpC20 grew from the solution of 1 M ammonium dihydrogen phosphate and 0.1 M n-(2-acetamido) iminodiacetic acid, pH 6.5. The best crystal of OmpC26 was grown from 0.1 M ammonium sulfate, 0.1 M 4-(2-hydroxyethyl)-1-piperazineethanesulfonic acid (HEPES)-sodium pH 7.5, 0.5 M di-sodium hydrogen phosphate dihydrate, and 0.5 M di-potassium hydrogen phosphate plus 0.1 M carbenicillin di-sodium. Crystals of OmpCO6 and OmpC20 were cryoprotected for data collection at 100 K by the addition of 20% glycerol to the mother liquor, OmpC26 by addition of 15% (v/v) glycerol. The best crystal of OmpC28 was grown from 0.1 M HEPES, pH 7.5, 0.15 M MgCl_2_, 21% (v/v) PEG400 plus 50 mM carbenicillin disodium at 20°C using 1% (w/v) βOG. The best crystal of OmpC33 was grown from 0.1 M HEPES, pH 7.5, 0.075 M ammonium sulfate, 22.5% (v/v) PEG400 at room temperature. Crystals were cryoprotected for data collection at 100 K by increasing the PEG400 concentration to 31% (v/v).

### Antibiotic transit

Plasmids pHSG575 OmpCK12, pHSG575 OmpC20, pHSG575 OmpC26, pHSG575 OmpC208 and pHSG575 OmpC33 transformed into *E. coli* Δomp8 cells [20] and MIC values determined as previously described [18]. Following a published protocol [18], cells were grown in liquid culture and the survival curves measured in the presence of antibiotics at various concentrations. The relative expression levels of the OmpC proteins were determined by normalizing cell density and then measuring the density of the OmpC band on SDS gels of the cell pellet and detergent extracted material. We found no difference in the expression level between the various OmpCs.

### Growth Experiments

Overnight cultures were grown at 37°C, 200 rpm, in LB medium supplemented with 12.5 µg ml^−1^ chloramphenicol as needed and then diluted into 20 ml fresh pre-warmed LB medium to an OD_650 nm_ of ∼0.05. Cells were grown to mid-exponential phase (OD_650 nm_∼0.4) and diluted 10-fold into 32.5 ml pre-warmed 5% (v/v) or 15% (v/v) LB medium. The 10-fold dilution of the LB pre-culture carries over 10% LB into the culture giving final concentrations of 15% (v/v) and 25% (v/v) LB. The OD_650_ was measured every 30 min.

### Cell viability assays

Overnight cultures were grown at 37°C, 200 rpm in Mueller Hinton broth (MHB) supplemented with 12.5 µg ml^−1^ chloramphenicol as needed and diluted into 20 ml fresh, pre-warmed MHB medium to an OD_650 nm_ of ∼0.05. Cells were grown to mid-exponential phase (OD_650 nm_∼0.4) and then diluted 10-fold into pre-warmed MHB medium containing one of 8 µg ml^−1^ cefotaxime, 2 µg ml^−1^ imipenem, 2 µg ml^−1^ ceftazidime and 2 µg ml^−1^ gentamicin (t = 0). Samples were taken at various time points and cell viability was determined by serial dilution of the samples to 10^−5^ in MHB medium and spotting 3×5 µl drops of each dilution onto MHB agar plates. Viable colonies were counted after 16 h incubation at 37°C and the number of cells ml^−1^ calculated. The results are expressed as % survival, where the cell ml^−1^ value at t = 0 is taken as 100%.

### MTT and MIC assay

The use of MTT to measure bacterial cell viability has been described previously [45]–[51]. NAD(P)H-dependent oxidoreductases and dehydrogenases of metabolically-active cells cleave the tetrazolium ring, yielding intracellular insoluble blue-magenta MTT formazan crystals. The amount of MTT formazan produced in a fixed time is proportional to the number of metabolically-active cells. Plasmids pHSG575 OmpC20 and pHSG575 OmpC33 transformed into HN705 cells were grown in 10 ml LB at 37°C to an OD600 of 1.0 before harvesting. As with the *E. coli* Δ8Omp cells, *E. coli* HN705 cells gave identical levels of expression of both OmpC proteins. The cell pellets were resuspended in PBS to give an OD_600_ of 0.4. A series of 10 µl antibiotic solutions in PBS or PBS alone were added to 90 µl of resuspended cells along with 10 µl of 5 mg ml^−1^ 3-[4,5-dimethylthiazol-2-yl]-2,5-diphenyl tetrazolium bromide (MTT, Sigma). After incubation for 4 h at 20°C the cells the formazan was extracted by the addition of 890 µl of acidified isopropanol. The absorbance of each assay was measured at wavelength of 570 nm with the background absorbance at 690 nm subtracted. A broad range of antibiotic concentrations was tested to identify the effective region, before detailed measurements in triplicate for each data point. The incubation without antibiotic for each OmpC was defined as 100% metabolic activity for that particular OmpC and inhibition expressed relative to this value. This normalization step controls for any variability in the uptake of MTT by the different OmpCs. Antibiotic and MTT are added simultaneously and the assay integrates over the whole population of cells for a fixed time period. The concentrations of antibiotics used in the assay are significantly above the MIC values, this is because it takes time for the effect of an antibiotic to be visible in a population of *E. coli* population. Since production of formazan is irreversible, up to the point the population becomes metabolically-inactive due to antibiotic pressure, formazan will accumulate. We found the results of this assay to be variable but the overall trend was consistent. The most divergent set of experiments are shown in Fig. S8. The route of entry of MTT into bacterial cells is not known but *E. coli* HN705 cells without the OmpC plasmid are able to produce similar amounts of formazan. The metabolic inhibition is expressed by: %inhibition = (A570c–A570s)/A570c *100; A570c is absorbance at 570 nm measured from cells with no antibiotic, A570s is absorbance at 570 nm measured from cells with added antibiotic.

MIC assays were performed in Mueller-Hinton broth according to the M.I.C.Evaluator strip guidelines (Oxoid). An overnight culture was diluted to an OD_600_ 0.15 (∼equivalent to MacFarland standard of 1) and 500 µl of this diluted culture was spread onto a Mueller-Hinton agar plate. Once the liquid had been absorbed by the agar plate, an M.I.C. Evaluator strip was placed flat onto the surface of the agar plate. The agar plate was incubated for 18 h at 37°C. The MIC values were read off the strip according to the manufacturer's instructions. The antibiotics tested were cefotaxime (32-0.002 µg ml^−1^), imipenem (32-0.002 µg ml^−1^) and gentamicin (256-0.016 µg ml^−1^).

### Cephalosporin uptake experiments

Cephalosporin uptake experiments were carried out with cefaloridine and cefazolin according to the published method [5], [26]. A single colony was used to inoculate 5 ml of LB, which was incubated, in a shaking incubator, overnight at 37°C. The overnight culture was diluted 1∶50 into pre-warmed LB containing 5 mM MgSO_4_. The culture was grown to about 100 Klett (∼OD650 = 0.8) and harvested. Subsequent procedures were conducted at 20°C, since incubation on ice can cause leakage of periplasmic proteins. The cell pellet was washed with 10 mM sodium phosphate buffer pH 6, containing 5 mM MgCl_2_ and resuspended in 3 ml of the same buffer (to give a concentration of 5 mg dry weight ml^−1^; 100 Klett = 0.3 mg dry weight ml^−1^). 0.5 ml was spun at 3,000 rpm for 10 min. The supernatant from this sample was retained and β-lactamase activity assayed to quantify any background activity due to cell disruption.

The assay mixture contained sodium phosphate buffer pH 6.0 containing 5 mM MgCl_2_ (420 µl), cell suspension (30 µl) (∼0.15 mg dry weight) and 10 mM cephalosporin (50 µl). Reagents and cells were mixed in a test tube and transferred to 1 mm path length cuvettes to minimize light scattering. The absorbance at 260 nm was measured and the assay repeated at least three times for each sample. Assays were performed with supernatant, with intact cells and sonicated cells. Sonicated cell extracts were prepared from the cell suspensions on ice (Sonic Dismembrator, Artek Systems Corp. Farmingdale, NY, USA, 50% power setting) by using four 30 s pulses separated by cooling periods. The assay of the extracts was repeated with different concentrations of cephalosporin to determine the concentration at which the hydrolysis rate was the same as that measured for the intact cells. The influx rate and permeability coefficient of the cells could then be calculated using the following equations:
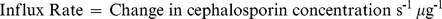


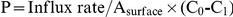


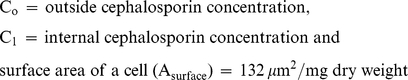



### Liposome Swelling Assay

The assay followed a published method [52]. 10 mM dicetylphosphate (20 µl) and 100 mM phosphatidylcholine (24 µl) were added to a clean, dry test-tube and dried under N_2_. Benzene (300 µl) was added to each tube then removed with N_2_ followed by anhydrous ether (500 µl), which was removed with N_2_. The tubes were then placed in a dessicator and the lipids dried under vacuum for at least an hour. The lipid film was resuspended in water (200 µl) (control) or water (200 µl) including 1–2 µg protein to be inserted into the liposome. The solution was vortexed and a sonic waterbath used to resuspend the lipid. The lipid-protein mixture was dried under vacuum. The dried protein/lipid film was resuspended in 10 mM Tris-HCl pH 8.0 (300 µl) containing 15% (w/v) dextran T-40 (Pharmacia) by slowly adding the later to the side of the test-tube and gently rotating the tube to wet the film. The tubes were left at room temperature for 30 min, shaken by hand and left for another 30 min.

The assay was carried out in 3 ml cuvettes and the change in absorbance at 400 nm measured. A 600 µl total volume contained: 60 µl 0.1 M Tris-HCl pH 8.0, x µl 0.1 M test solute (where x was varied to establish iso-osmolarity). The components were mixed in the cuvette, proteoliposome (17 µl) was added and readings were taken immediately. Control measurements to establish isotonic conditions were carried out with liposomes lacking the porin by incubation with different concentrations of stachyose (a solute which is impermeant to *E. coli* porins) to find a concentration that caused neither swelling nor shrinking. The assay was repeated with the other test solutes to ensure they were isotonic. Having established these core conditions, the porin-containing liposomes were assayed in triplicate with iso-osmotic concentrations of solute using a variety of test solutes with differing molecular weights (arabinose, glucose, N-acetyl-glucosamine). The permeation rate was then calculated as the change in absorbance min^−1^ (µg^−1^ of protein) which reflects liposome swelling.

### Single channel conductance experiments

Single-channel recordings were carried out in solvent-free planar lipid membranes [53]. Briefly, a 25-µm-thick Teflon film (Goodfellow) with a 100 µm diameter orifice partitioned two 1.5 ml chambers. The orifice was pretreated with ∼1 µl 1% (v/v) hexadecane dissolved in high-purity pentane. Each chamber was filled with 1 ml of 10 mM Tris-HCl, pH 8.0, 1 M KCl. Lipid solution in pentane (10 µl, 4 mg ml^−1^) was added to each chamber and a monolayer formed after pentane evaporation. By lowering and raising the solution, a lipid bilayer was formed at the orifice, confirmed by its resistance and capacitance. For single channel measurements, 1,2-diphytanoyl-sn-glycero-3-phosphocholine (Avanti Polar Lipids) (DPhPC) was used. For sequential multi-channel insertions, DPhPC with 10 mol% cholesterol and 2 mol% 1,2-distearoyl-sn-glycero-3-phosphocholine (DSPC) (both Avanti Polar Lipids) was substituted to facilitate channel reconstitution at low voltages. Transmembrane potential was applied with two Ag/AgCl electrodes. The cis chamber corresponds both to electrical ground and the side of protein addition. Experiments were conducted at room temperature.

Single-channel currents were amplified with an Axopatch 200B patch-clamp amplifier (Axon Instruments), and filtered at 1 kHz with a built-in low-pass 4-pole Bessel filter. Signals were digitized with a Digidata 1440 A/D converter at a sampling rate of 10 kHz and recorded by Clampex 10.2 software (Axon). The data were analyzed with the software pClamp 10.2 (Axon) and Origin 7.0 (Microcal Software Inc.). The insertion of a single trimeric channels into the bilayer was facilitated by applying electrical potentials of +250 to +300 mV, after addition of purified OmpC in 1% βOG to a final concentration of 10 ng ml^−1^. Channels were stabilized for recording by lowering the voltage to below +250 mV. For continuous multi-channel insertions, final concentrations up to 0.5 µg ml^−1^ were used.

### Structural biology

Data were collected at the European Synchrotron Radiation Facility (Table 2), indexed and integrated using MOSFLM [54]. For OmpC26 multiple lattices were evident and the strongest lattice within the resolution range 3 Å - 2.3 Å was selected for indexing. POINTLESS [55] XTRIAGE [56] were used to assess quality and assign the correct space group; data were merged with SCALA [57]. Ellipsoidal truncation was performed for data of OmpC26 [58] and this reduced completeness by 20%.

All the structures were solved using molecular replacement with PHASER [59], [60] or MOLREP [61]. The structure of OmpCO6 used OmpK36 from *Klebsiella pneumoniae* (PDB: 1OSM) as a model. Subsequently OmpCO6 was used as the search model for the other structures. Refinement and model building were carried out using REFMAC5 [62] and Coot [63]. NCS restraints and TLS refinement were employed. The stereochemical quality was analyzed using MolProbity [64] and figures produced using PYMOL (www.pymol.org).

### Molecular Dynamics

We prepared OmpC20 starting from its X-ray structure. We inserted the porin in a previously relaxed POPC bilayer where after removal of overlapping molecules, we arrived to a system with 215 POPC and solvated with 18582 water molecules (hexagonal cell 107×107×96 Å). We relaxed the system slowly to 300 K (1.5 ns) maintaining both the position of the protein restrained to the X-ray structure and the position of the POPC headgroup fixed along the axis Z, to avoid the opening of the bilayer. After releasing the restraint on POPC we relaxed for 4 ns before neutralizing the box with the addition of counter ions (Na^+^). An additional relaxation of 10 ns was followed by 45 ns simulations that we used for structural analysis. We considered this long enough to properly sample the protein-protein interactions. Due to the lower quality of OmpC33 X-ray structure, we decided to start with the OmpC28 X-ray coordinates performing the substitution R124H. Then we followed the same protocol as above. We used the Amber99SB force field as before [65]. These simulations were performed with the software NAMD, version 2.7.

We performed metadynamics simulations to investigate the translocation of the anionic cephalosporin cefotaxime using the program ORAC [66]. We set-up the antibiotic force field as described [65] and we placed it in the relaxed structure of OmpC20 and OmpC33 at the extracellular mouth channel, approximately 14 Å from the constriction region. As before [65], [67], [68] enhanced sampling is used to ensure the simulation accesses all the relevant conformational states of the antibiotic inside the channels. On the basis of earlier findings [65], [67], [68] we chose two relevant collective variables to bias the translocation of cefotaxime: (i) the variable *Z*, defined as the difference between the center of mass of the antibiotic and the center of mass of monomer 1 along the *z*-axis, the axis diffusion; and (ii) the angle θ, which defines the orientation of the long axis of the molecule with respect to the *z*-axis. A Gaussian potential was added every 4 ps with a height of 0.48 kcal mol^−1^ and width of 0.4 Å and 5.0°, respectively, for the coordinates *Z* and θ. These parameters were chosen to allow a better resolution in the sampling of the free energy surface (FES) in a simulation time of 27 ns and 31 ns, respectively for OmpC20 and OmpC33. We estimated the total error of our simulation strategy to be around 2 kcal mol^−1^ [68]. When cefotaxime penetrates with the aminothiazoly group the coordinate θ has a value near 180°, correspondingly it is near 0° in the opposite orientation.

Due to the complexity of the process studied, we reconstructed the free energy surface from metadynamics simulations after obtaining the first and only translocation path, as done elsewhere [31], [68], [69]. This first crossing is considered to be the most probable path because it passes through the lowest saddle point. The barrier is calculated as the difference between the lowest minimum in the extracellular side and the saddle point after passing the constriction region. This barrier, being the highest, controls the flux of molecules, lower the barrier higher the flux.

## Supporting Information

Figure S1 Sequence alignment of OmpC from clinical isolates^1^ and OmpCO6 from uropathogenic *E. coli* O6:H1 (accession no. Q8CVW1). The alignment was carried out in Multalin. Above the alignment, the β-strands forming the barrel and the extracellular loops connecting the β-strands are numbered. The β-strands are labelled β1–β16 and β1′. Strand β1 is from the N-terminal and strand β1′ is from the C-terminal are two parts of the same strand. The residue numbering is according to that of OmpCO6. The mutation sites between the clinical OmpC mutants are highlighted in red.
(TIFF)

Figure S2 Expression of the cloned OmpC mutants in Δ8omp (a) and HN705 (b) cells. (a) From left to right, Lane 1 is SeeBlue marker and lanes 2–7 are whole cell protein samples corresponding to Δ8omp strain no plasmid, Δ8omp strain expressing OmpC K12, OmpC20, OmpC26, OmpC28 and OmpC33 respectively. (b) From left to right, Lane 1,2,3 are whole cell protein, corresponding to HN705 knocked out strain, HN705 expressing OmpC20, and HN705 expressing OmpC33, respectively; lane 4,5,6 are outer membrane extractions of HN705 no plasmid, cells expressing OmpC20 and OmpC33 respectively. Mass-spec analysis showed that the major band in Lane 4 is PhoE which appears upregulated in the absence of OmpC. ImageGauge showed that the intensity of gel bands 5 and 6 were the same, indicating the same expression level of OmpC20 and OmpC33. **Experimental details:** (a) Cells were grown to OD_650_ 0.4, harvested and resuspended in 20 µl SDS solubilisation buffer (20% (v/v) glycerol, 2% (w/v) SDS, 0.12 M Tris-HCl, pH 6.8, 0.5 g L^−1^ bromophenol blue, 20 ml L^−1^ β-mercaptoethanol) heated at 100°C for 10 min, centrifuged and the gel loaded with the supernatant (15 µl). (b) Lanes1–3: Cells were grown to OD_600_ 1.0 and washed cells (20 µl) (wash buffer PBS, pH 7.4) were incubated with 10 µl NuPAGE loading buffer (Invitrogen) heated at 100°C for 10 min, centrifuged and the gel loaded with the supernatant (18 µl). Lanes 4–6: 0.5 L of cells grown to OD_600_ 1.0 harvested, disrupted and membranes collected by centrifugation at 100,000 g. Protein extraction followed the same procedure as for protein purification (see methods). After SB3.14 extraction, 20 µl outer membrane protein crude solution was incubated with 10 µl NuPAGE loading buffer (Invitrogen) heated at 100°C for 10 min, centrifuged and the gel loaded with the supernatant (15 µl). Camera and software: FUJIFILM ImageReader LAS-1000 Pro V2.3. Software: FUJIFILM ScienceLab 2003, ImageGauge V4.21.
(TIF)

Figure S3 Evidence that mutation in OmpC affects cell viability. (a) LB growth curve: Δomp9 cells transformed with the empty vector (navy squares), OmpCK12 (magenta diamonds) OmpC20 (purple squares), OmpC26 (green triangles), OmpC28 (cyan diamonds) and OmpC33 (red circles) were grown in LB medium to mid-exponential phase and then diluted 1 in 10 into 15% diluted LB medium to give a final 25% strength LB culture. A representative example of three replicates is shown. (b–d) Viability assays were performed with 2 µg.ml^−1^ imipenem (b) 4 µg.ml^−1^ ceftazidime (c) 2 µg.ml^−1^ gentamicin (d) of ΔOmp8 cells transformed with the empty vector pHSG575, OmpC K12, OmpC20, OmpC26, OmpC28 and OmpC33 (colours as (a)). The mean ± SD of a minimum of 3 replicates are shown. (e) Gentamicin susceptibility testing of each of the four clinical isolates (open bars) in parallel with these same isolates transformed with a plasmid carrying the *ompC* K12 gene (grey bars) was performed using M.I.C.Evaluator strips in Mueller-Hinton broth.
(TIF)

Figure S4 (a) Superposition of OmpC20 (cyan), *E. coli* OmpC K12 (ochre)and OmpCO6 (pink) In (a) Trimer shown looking into the constriction zone towards the periplasm and (b) monomer shown with periplasm at bottom and extracellular region at top (almost 90° rotation from (a)). The loops L2, L3 and L4 are labeled in the one monomer. The main differences in the structures occur in L4 which folds across the timer interface and forms the entrance to the constriction zone. OmpC20 has a shorter loop. L2 which folds across the trimer interface in the neighboring monomer and L3 which forms the constriction zone are essentially identical. There are smaller differences in the other extracellular loops. (c) Radius of central channel through the different OmpC's crystal structures, calculated by program HOLE [30]. The left hand side of the graph corresponds to the periplasmic face (bottom of S4b) and the right hand side to the extracellular space (top of S4b). The constriction zone is about half down the 28 Å long central channel. (d) Area available to a probe of radius 1.4 A: averaged over 20 ns MD simulation trajectories at T = 300 K and P = 1 Atm.
(TIF)

Figure S5 Electrostatic surface of OmpC. (a) OmpC K12 (b) OmpCO6, (c) OmpC20 (d) OmpC26 (e) OmpC28 (f) OmpC33. Pictures were generated in CCp4MG with the same parameters. Blue is positive charge and red is negative charge. The view is looking down into the constriction zone (from outside the cell into the cell). The entrance to the pore is less occluded (shorter L4) and more negatively charged in OmpC20 (26, 28 and 33) than in either OmpC K12 or OmpCO6.
(TIF)

Figure S6 Comparison of thermal stability between OmpC's. In common with other trimeric porins, the folded OmpC trimer although runs at a higher weight its runs close to the weight of the monomer. (a) OmpC20 and OmpC33 were purified in 1% BOG detergent and heated at a range of temperatures marked in °C. (b) All clinically derived strains proteins were purified in the detergent SB 3.14 detergent and heated to various temperatures. OmpC33 is notably more prone to dissociation into monomers than the other proteins.
(TIF)

Figure S7 Interactions between antibiotic and pore. (**a**) The lifetime of hydrogen bond interactions between cefotaxime atoms and OmpC20 residue atoms. (b) The lifetime of hydrogen bond interactions between cefotaxime atoms and OmpC33 residue atoms. The simulations were performed as described in the paper.
(TIF)

Figure S8 The most divergent repeat of the antibiotic inhibition experiment. A different batch of cells and reagents were used. Although the details of the results vary from Figure 2c, the trend (OmpC33 more resistant) is consistent.
(TIF)
