# Supplementary figures and images for: Altered Antibiotic Transport in OmpC Mutants Isolated from a Series of Clinical Strains of Multi-Drug Resistant E. coli
Source: PLoS One. 2011 Oct 28;6(10):e25825. doi: 10.1371/journal.pone.0025825 (PMC3203869; doi:10.1371/journal.pone.0025825)

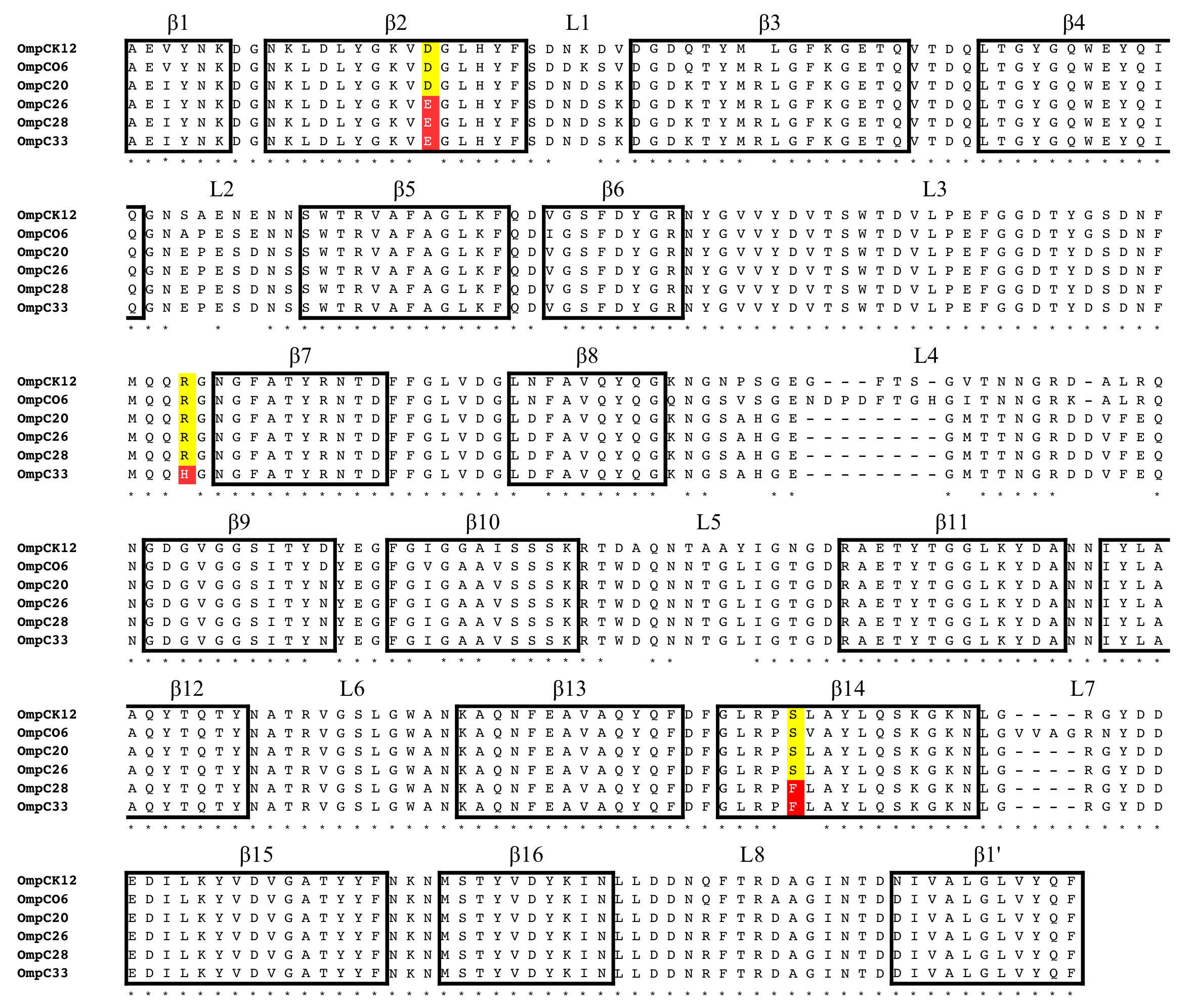

Supplement: Figure S1 — Sequence alignment of OmpC from clinical isolates1 and OmpCO6 from uropathogenic E. coli O6:H1 (accession no. Q8CVW1). The alignment was carried out in Multalin. Above the alignment, the β-strands forming the barrel and the extracellular loops connecting the β-strands are numbered. The β-strands are labelled β1–β16 and β1′. Strand β1 is from the N-terminal and strand β1′ is from the C-terminal are two parts of the same strand. The residue numbering is according to that of OmpCO6. The mutation sites between the clinical OmpC mutants are highlighted in red. (TIFF) [file pone.0025825.s001.tiff]

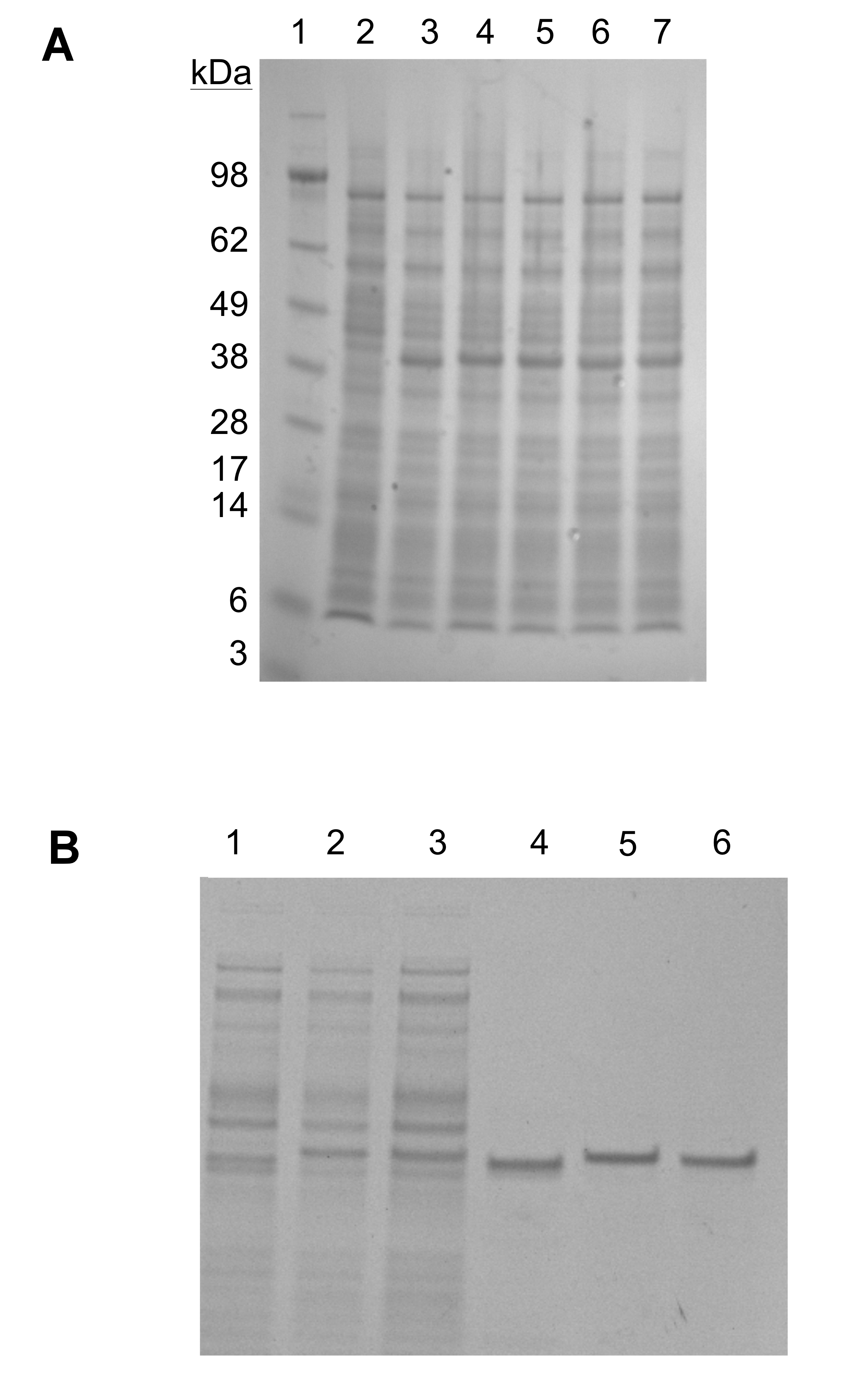

Supplement: Figure S2 — Expression of the cloned OmpC mutants in Δ8omp (a) and HN705 (b) cells. (a) From left to right, Lane 1 is SeeBlue marker and lanes 2–7 are whole cell protein samples corresponding to Δ8omp strain no plasmid, Δ8omp strain expressing OmpC K12, OmpC20, OmpC26, OmpC28 and OmpC33 respectively. (b) From left to right, Lane 1,2,3 are whole cell protein, corresponding to HN705 knocked out strain, HN705 expressing OmpC20, and HN705 expressing OmpC33, respectively; lane 4,5,6 are outer membrane extractions of HN705 no plasmid, cells expressing OmpC20 and OmpC33 respectively. Mass-spec analysis showed that the major band in Lane 4 is PhoE which appears upregulated in the absence of OmpC. ImageGauge showed that the intensity of gel bands 5 and 6 were the same, indicating the same expression level of OmpC20 and OmpC33. Experimental details: (a) Cells were grown to OD650 0.4, harvested and resuspended in 20 µl SDS solubilisation buffer (20% (v/v) glycerol, 2% (w/v) SDS, 0.12 M Tris-HCl, pH 6.8, 0.5 g L−1 bromophenol blue, 20 ml L−1 β-mercaptoethanol) heated at 100°C for 10 min, centrifuged and the gel loaded with the supernatant (15 µl). (b) Lanes1–3: Cells were grown to OD600 1.0 and washed cells (20 µl) (wash buffer PBS, pH 7.4) were incubated with 10 µl NuPAGE loading buffer (Invitrogen) heated at 100°C for 10 min, centrifuged and the gel loaded with the supernatant (18 µl). Lanes 4–6: 0.5 L of cells grown to OD600 1.0 harvested, disrupted and membranes collected by centrifugation at 100,000 g. Protein extraction followed the same procedure as for protein purification (see methods). After SB3.14 extraction, 20 µl outer membrane protein crude solution was incubated with 10 µl NuPAGE loading buffer (Invitrogen) heated at 100°C for 10 min, centrifuged and the gel loaded with the supernatant (15 µl). Camera and software: FUJIFILM ImageReader LAS-1000 Pro V2.3. Software: FUJIFILM ScienceLab 2003, ImageGauge V4.21. (TIF) [file pone.0025825.s002.tif]

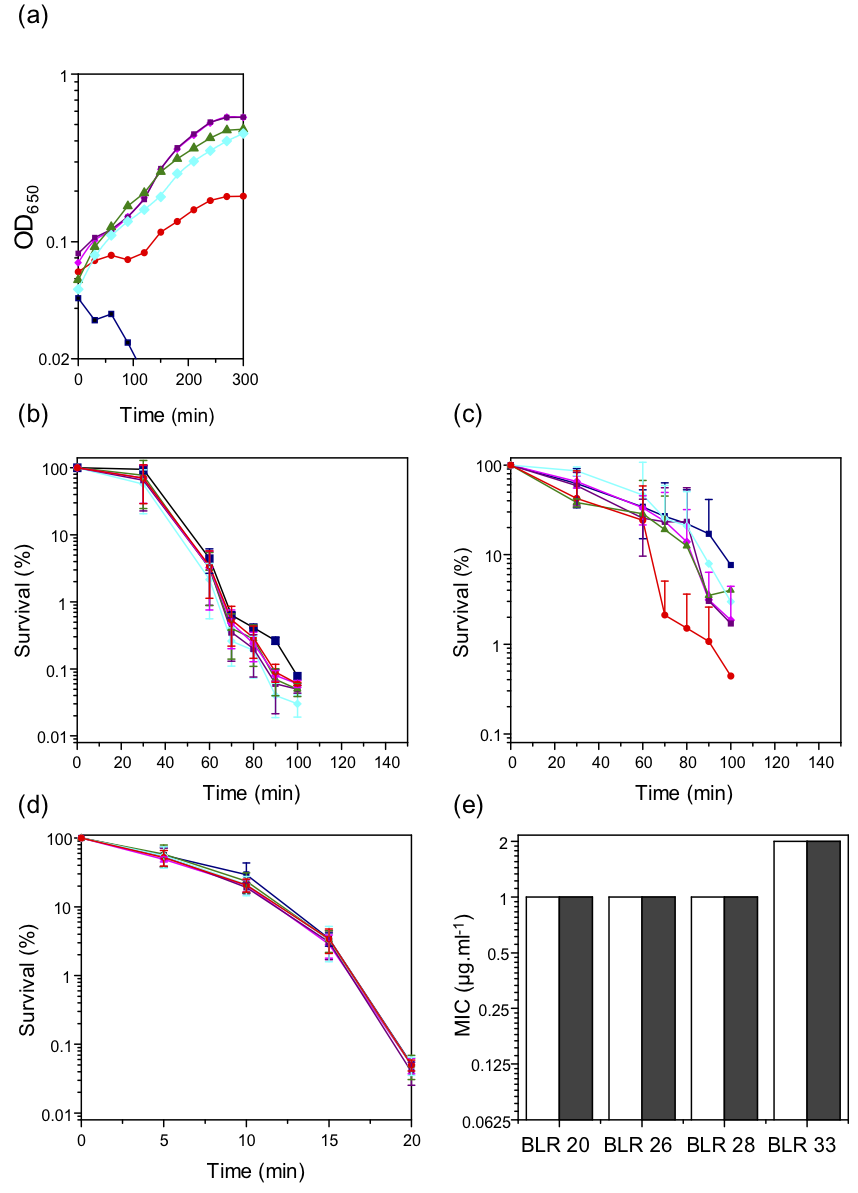

Supplement: Figure S3 — Evidence that mutation in OmpC affects cell viability. (a) LB growth curve: Δomp9 cells transformed with the empty vector (navy squares), OmpCK12 (magenta diamonds) OmpC20 (purple squares), OmpC26 (green triangles), OmpC28 (cyan diamonds) and OmpC33 (red circles) were grown in LB medium to mid-exponential phase and then diluted 1 in 10 into 15% diluted LB medium to give a final 25% strength LB culture. A representative example of three replicates is shown. (b–d) Viability assays were performed with 2 µg.ml−1 imipenem (b) 4 µg.ml−1 ceftazidime (c) 2 µg.ml−1 gentamicin (d) of ΔOmp8 cells transformed with the empty vector pHSG575, OmpC K12, OmpC20, OmpC26, OmpC28 and OmpC33 (colours as (a)). The mean ± SD of a minimum of 3 replicates are shown. (e) Gentamicin susceptibility testing of each of the four clinical isolates (open bars) in parallel with these same isolates transformed with a plasmid carrying the ompC K12 gene (grey bars) was performed using M.I.C.Evaluator strips in Mueller-Hinton broth. (TIF) [file pone.0025825.s003.tif]

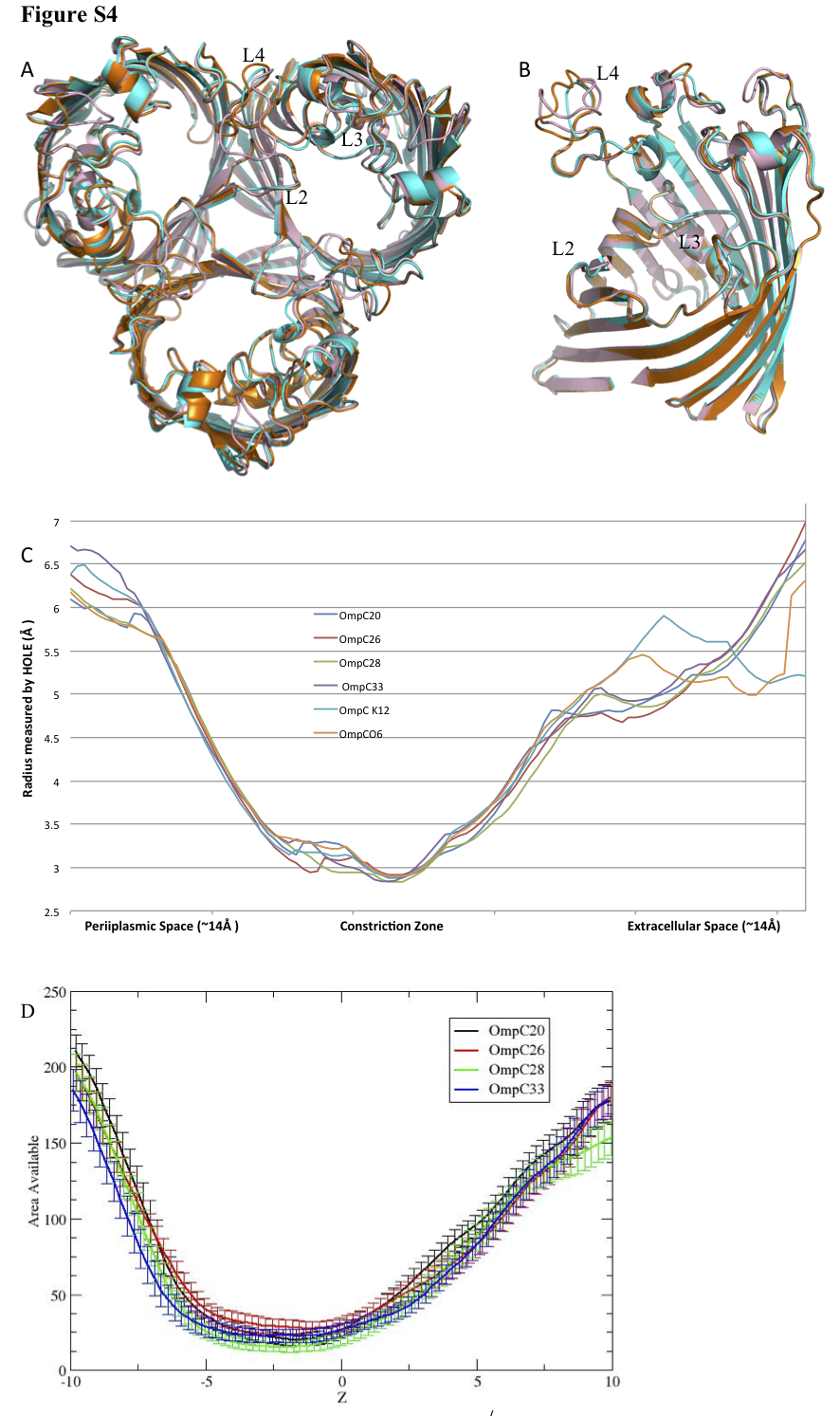

Supplement: Figure S4 — (a) Superposition of OmpC20 (cyan), E. coli OmpC K12 (ochre)and OmpCO6 (pink) In (a) Trimer shown looking into the constriction zone towards the periplasm and (b) monomer shown with periplasm at bottom and extracellular region at top (almost 90° rotation from (a)). The loops L2, L3 and L4 are labeled in the one monomer. The main differences in the structures occur in L4 which folds across the timer interface and forms the entrance to the constriction zone. OmpC20 has a shorter loop. L2 which folds across the trimer interface in the neighboring monomer and L3 which forms the constriction zone are essentially identical. There are smaller differences in the other extracellular loops. (c) Radius of central channel through the different OmpC's crystal structures, calculated by program HOLE [30]. The left hand side of the graph corresponds to the periplasmic face (bottom of S4b) and the right hand side to the extracellular space (top of S4b). The constriction zone is about half down the 28 Å long central channel. (d) Area available to a probe of radius 1.4 A: averaged over 20 ns MD simulation trajectories at T = 300 K and P = 1 Atm. (TIF) [file pone.0025825.s004.tif]

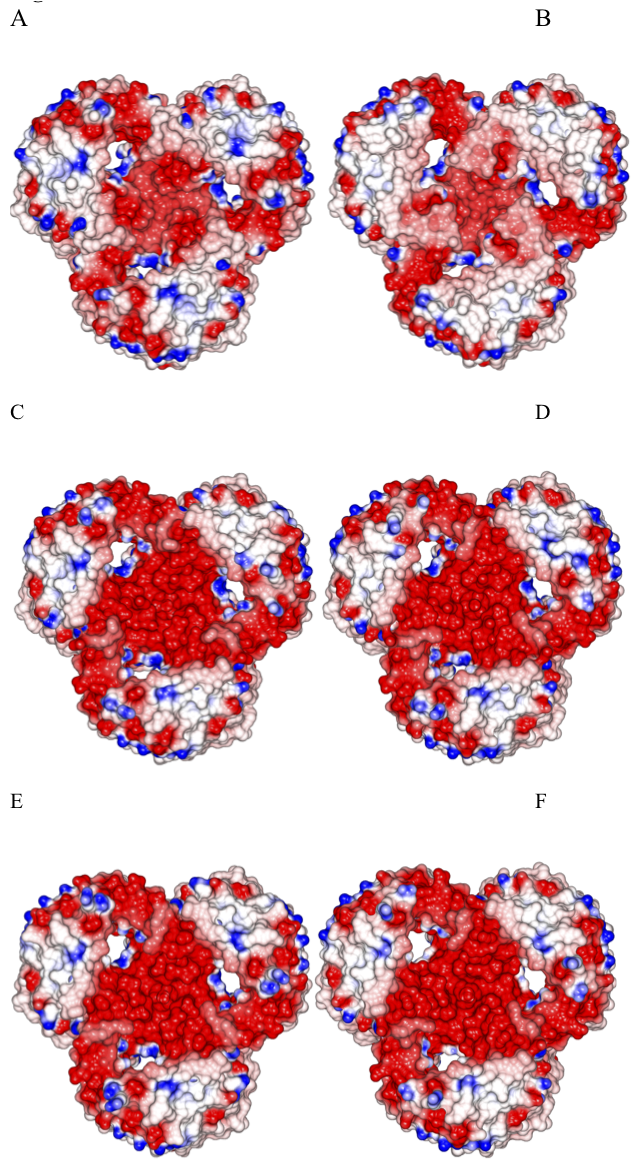

Supplement: Figure S5 — Electrostatic surface of OmpC. (a) OmpC K12 (b) OmpCO6, (c) OmpC20 (d) OmpC26 (e) OmpC28 (f) OmpC33. Pictures were generated in CCp4MG with the same parameters. Blue is positive charge and red is negative charge. The view is looking down into the constriction zone (from outside the cell into the cell). The entrance to the pore is less occluded (shorter L4) and more negatively charged in OmpC20 (26, 28 and 33) than in either OmpC K12 or OmpCO6. (TIF) [file pone.0025825.s005.tif]

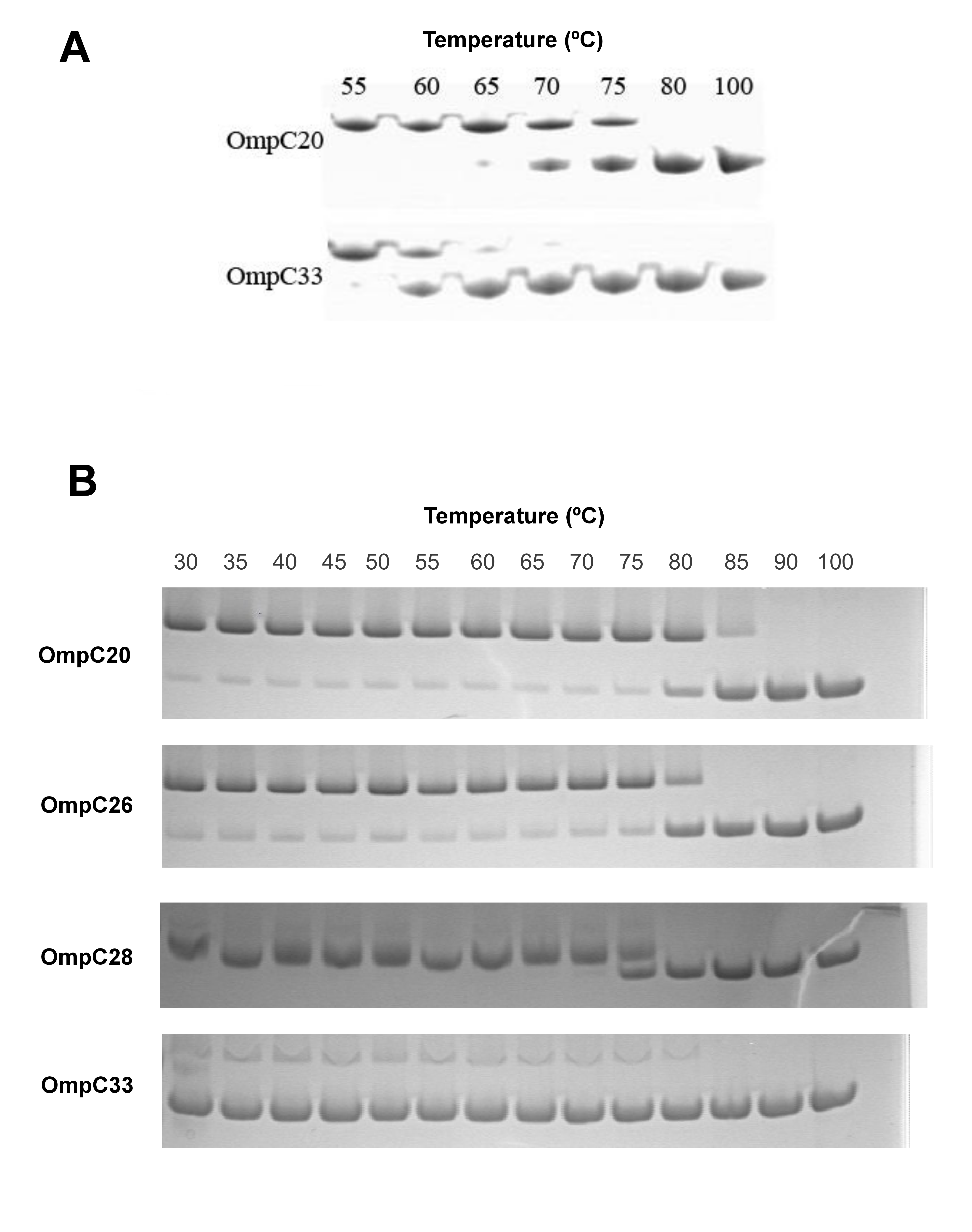

Supplement: Figure S6 — Comparison of thermal stability between OmpC's. In common with other trimeric porins, the folded OmpC trimer although runs at a higher weight its runs close to the weight of the monomer. (a) OmpC20 and OmpC33 were purified in 1% BOG detergent and heated at a range of temperatures marked in °C. (b) All clinically derived strains proteins were purified in the detergent SB 3.14 detergent and heated to various temperatures. OmpC33 is notably more prone to dissociation into monomers than the other proteins. (TIF) [file pone.0025825.s006.tif]

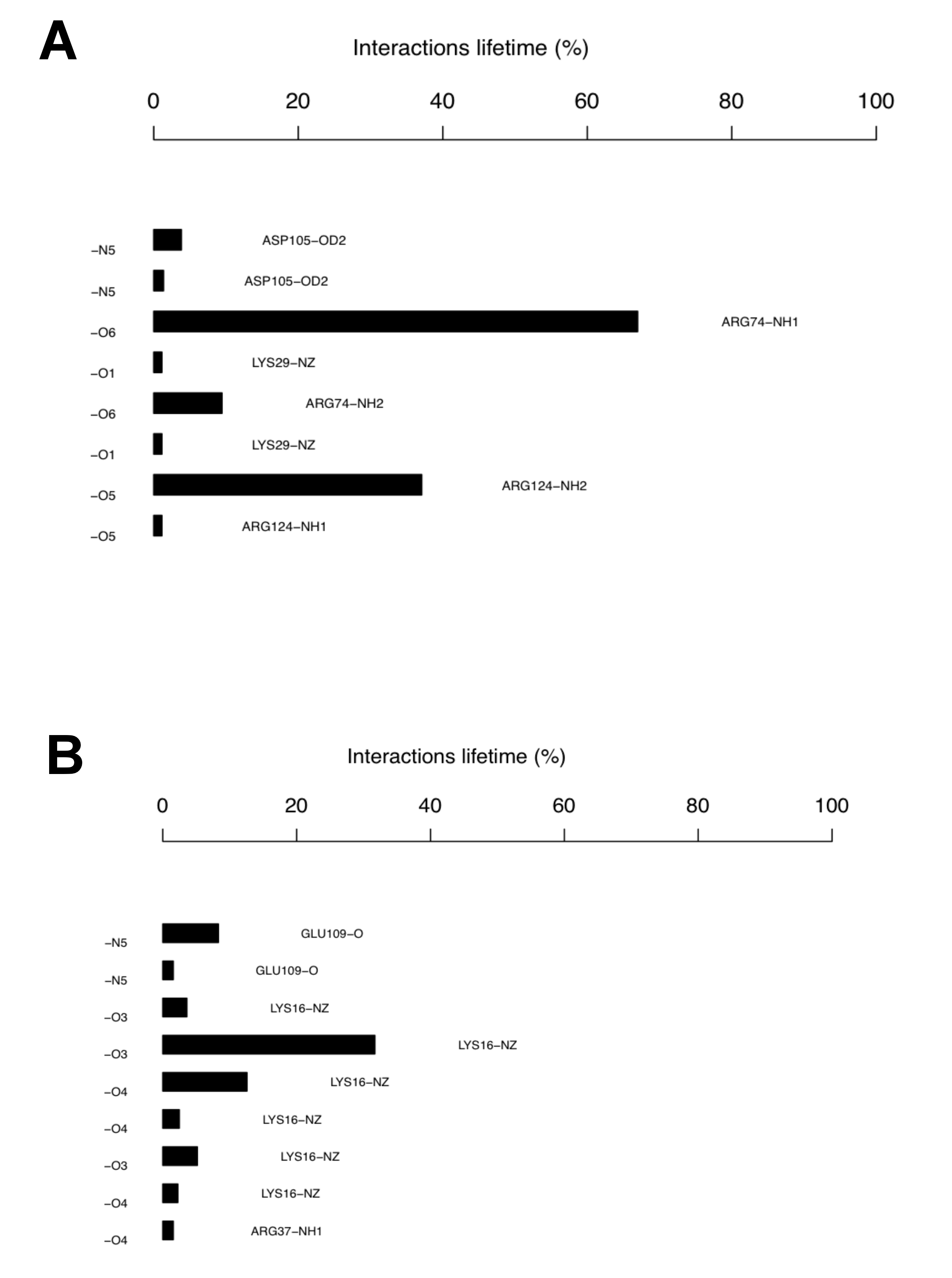

Supplement: Figure S7 — Interactions between antibiotic and pore. (a) The lifetime of hydrogen bond interactions between cefotaxime atoms and OmpC20 residue atoms. (b) The lifetime of hydrogen bond interactions between cefotaxime atoms and OmpC33 residue atoms. The simulations were performed as described in the paper. (TIF) [file pone.0025825.s007.tif]

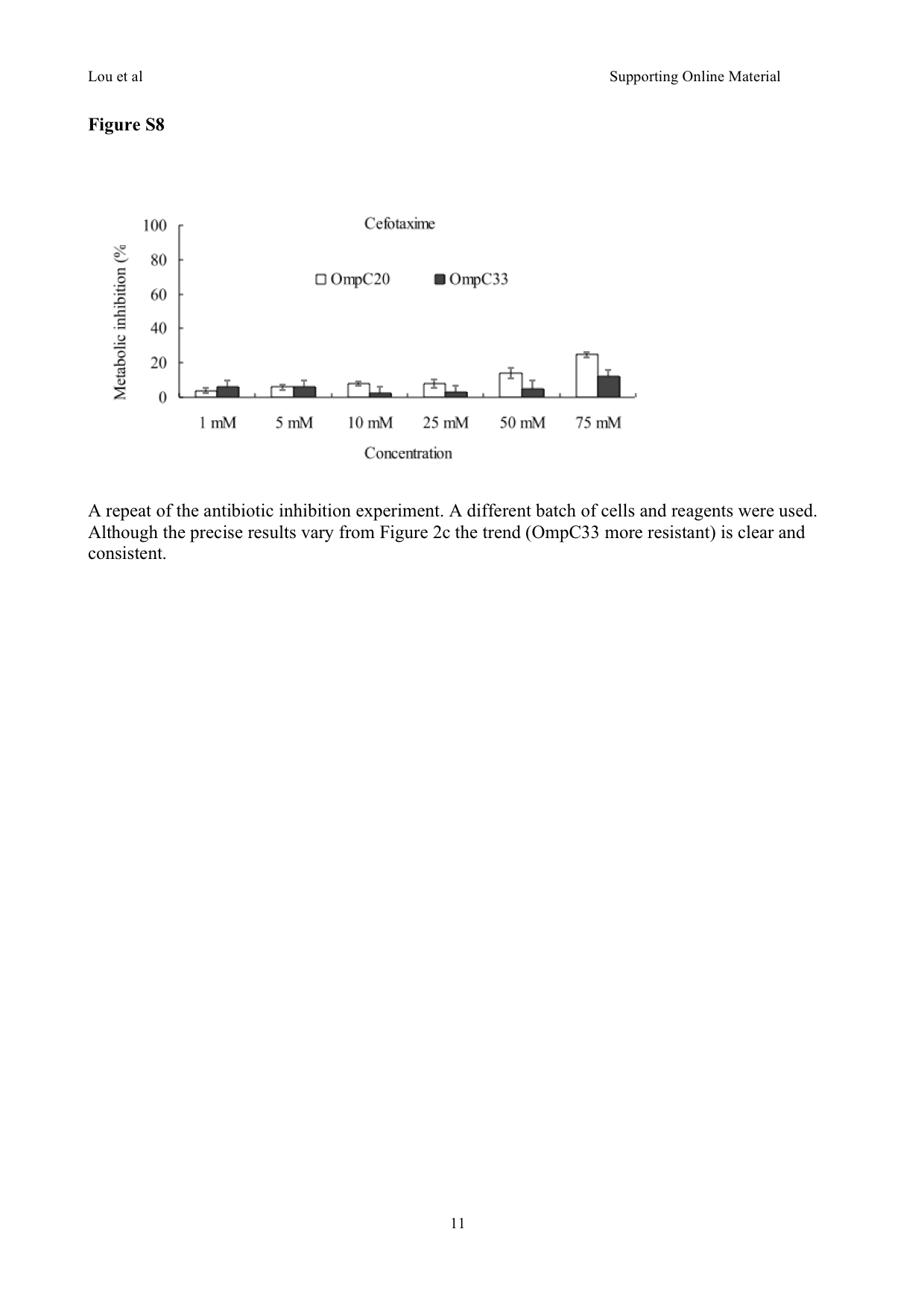

Supplement: Figure S8 — The most divergent repeat of the antibiotic inhibition experiment. A different batch of cells and reagents were used. Although the details of the results vary from Figure 2c, the trend (OmpC33 more resistant) is consistent. (TIF) [file pone.0025825.s008.tif]
